# Anlotinib enhances the sensitivity to docetaxel in lung cancer through inhibiting DDR1-mediated glycolytic pathway

**DOI:** 10.7150/ijbs.129604

**Published:** 2026-08-24

**Authors:** Xueqin Chen, Juan Shen, Zhifei Xu, Bo Xu, Jie Huang, Jingjing Jiang, Yidan Chen, Xin Li, Shaoyu Yang, Kan Wu, Jiaoli Wang, Peihua Luo, Qiaojun He, Shenglin Ma, Bing Xia, Bo Yang

**Affiliations:** 1 Department of Thoracic Oncology, Affiliated Hangzhou First People's Hospital, School of Medicine, Westlake University, Hangzhou, Zhejiang 310006, PR China.; 2 Department of Thoracic Oncology, Hangzhou Cancer Hospital, Hangzhou, Zhejiang 310002, PR China.; 3 Fourth Clinical Medical College, Zhejiang Chinese Medical University, Hangzhou, Zhejiang 310053, PR China.; 4 Center for Drug Safety Evaluation and Research of Zhejiang University, College of Pharmaceutical Sciences, Zhejiang University, Hangzhou, Zhejiang 310058, PR China.; 5 Innovation Institute for Artificial Intelligence in Medicine of Zhejiang University, Hangzhou, Zhejiang 310018, PR China.; 6 School of Medicine, Hangzhou City University, Hangzhou, Zhejiang 310015, PR China.; 7 School of Medicine, Westlake University, Hangzhou, Zhejiang 310024, PR China.; 8 Institute of Pharmacology & Toxicology, College of Pharmaceutical Sciences, Zhejiang University, Hangzhou, Zhejiang 310058, PR China.

**Keywords:** DDR1, anlotinib, docetaxel, glycolysis, sensitivity

## Abstract

Discoidin domain receptor 1 (DDR1), a collagen-activated receptor tyrosine kinase, is essential for tumor cell proliferation, invasion and drug resistance. Our study demonstrated that the expression and phosphorylation of DDR1 were associated not only with the poor outcome of non-small cell lung cancer (NSCLC), but also with low sensitivity to docetaxel. Moreover, our kinase profiling identified that anlotinib, an oral small-molecule tyrosine kinase inhibitor, could significantly inhibit DDR1 phosphorylation. Further data revealed that anlotinib could increase the sensitivity to docetaxel in cell lines, cell-derived and patient-derived tumor xenografts through down-regulating DDR1 phosphorylation, which no longer existed in *DDR1*-KO A549 cells. Mechanistically, anlotinib could enhance the sensitivity to docetaxel through inhibiting DDR1-mediated glycolysis. Additionally, the clinical trial demonstrated that anlotinib could increase the efficacy of docetaxel, with an improved overall response rate of 35.6%, a prolonged median progression-free survival by 5.3 months and a median overall survival by 14.0 months compared with historical data. In summary, our study not only highlights the new function of DDR1 in energy metabolism but also provides further evidence for the combination of anlotinib and docetaxel as a preferable strategy in clinical practice.

## Introduction

Lung cancer remains the most prevalent malignant tumor in terms of both incidence and mortality in 2022, globally and in China 1,2. Non-small cell lung cancer (NSCLC) accounts for 85%-90% of malignant lung neoplasms, with approximately 75% of NSCLC already at stage IIIB or IV at the time of diagnosis 3. For patients with metastatic NSCLC without oncogenic drivers or those with oncogenes but progressing on molecular targeted therapy, platinum-doublet chemotherapy combined with or without immune checkpoint inhibitors or antiangiogenic agents is the standard therapeutic regimen. Although these combined treatments have improved clinical outcomes, eventual disease progression or treatment failure remains an inevitable challenge.

Compared with the best supportive care, docetaxel has been approved as standard second- or later-line therapy for advanced NSCLC on the basis of improved survival, regardless of histology or gene status 4,5. However, its clinical outcomes are relatively poor, with an overall response rate (ORR) of less than 10%, median progression-free survival (PFS) of approximately 3 months and median overall survival (OS) ranging from 7.0 to 9.6 months 4-7. Moreover, other similar chemical agents, such as pemetrexed, have been shown to be non-inferior to docetaxel in terms of survival 7. To improve the efficacy of treatment and the survival of patients, clinical trials of docetaxel combined with nintedanib or with ramucirumab have been conducted 8,9. However, the benefits are limited and nintedanib and ramucirumab are not approved for lung cancer in China. Therefore, the need to improve clinical efficacy of docetaxel has emerged as an urgent clinical issue to address.

Discoidin domain receptor 1 (DDR1), a collagen-activated tyrosine kinase receptor, is essential for tumor cell proliferation, differentiation, invasion and migration across various malignancies 10. Additionally, the expression level of DDR1 was correlated with poor response or drug resistance in lung cancer 11-16. However, whether DDR1 is involved in regulating sensitivity to docetaxel remains unknown, and there are still no available small-molecule DDR1 inhibitors for clinical application.

Anlotinib, an oral small-molecule tyrosine kinase inhibitor that targets multiple kinases including vascular endothelial growth factor receptor (VEGFR)1/2/3 and fibroblast growth factor receptor (FGFR)1/2/3, can effectively suppress both tumor angiogenesis and tumor cell proliferation 17-20. A randomized, double-blind, multicenter, placebo-controlled phase Ⅲ clinical trial demonstrated that anlotinib could significantly prolong OS and PFS and was well tolerated in advanced NSCLC patients who had failed at least two lines of systemic chemotherapy 18. Recently, a phase Ⅱ trial revealed that the addition of anlotinib to docetaxel could increase anti-tumor efficacy and prolong PFS in patients with advanced NSCLC who experienced failure with first-line platinum-based chemotherapy 21. Our kinase profile screening study demonstrated that anlotinib could potently inhibit DDR1, and this inhibition of DDR1 by anlotinib has potential to increase the efficacy of docetaxel.

In this study, we demonstrated that anlotinib could enhance the anti-tumor effects of docetaxel in NSCLC, as evidenced by consistent results from cellular experiments, animal models and clinical trial. Mechanistically, the increased level of DDR1 phosphorylation contributed to the diminished sensitivity to docetaxel, while anlotinib, a DDR1 inhibitor, could increase the sensitivity to docetaxel by targeting DDR1-mediated glycolysis pathway. In summary, our study provides evidence and a theoretical basis for the combination of anlotinib and docetaxel as a meaningful strategy to improve the clinical efficacy of docetaxel in patients with NSCLC.

## Materials and Methods

### Cell lines and mice

The human non-small cell lung cancer cell lines A549, PC9, and H1299 were obtained from the Typical Culture Collection Committee of the Chinese Academy of Sciences (Shanghai, China). These cells were cultured in RPMI-1640 medium supplemented with 10% fetal bovine serum at 37 °C in a humidified atmosphere of 95% air and 5% CO_2_.

BALB/c nude mice were used to construct subcutaneous xenograft tumors, and NOD-SCID mice were used to construct PDX models as previously described 22. Male BALB/c nude mice and NOD-SCID mice (both 3-5 weeks old) were purchased from SLAC Laboratory Company (Shanghai, China). All the mice were housed under a 12-h light/dark cycle with sufficient food and water. All experimental procedures were performed in accordance with animal ethical standards and were conducted in accordance with the guidelines of the Institutional Animal Care and Use Committee (IACUC), and were approved by the Drug Safety Evaluation Research Center of Zhejiang Chinese Medical University (approval numbers: IACUC-20181210-01, IACUC-20221008-05 and IACUC-20250120-14).

### Formalin-fixed paraffin-embedded (FFPE) tumor samples

FFPE tumor samples from 12 NSCLC patients were obtained from Hangzhou Cancer Hospital and Affiliated Hangzhou First People's Hospital, School of Medicine, Westlake University. Additionally, baseline characteristics and follow-up survival data of these patients were gathered. The use of these specimens and patient information was approved by the Ethics Committee of Hangzhou Cancer Hospital (approval number: HZCH-2025-FTR-001).

### Gene editing

Knockdown of *DDR1*. NSCLC cells were transfected with siRNA oligonucleotides using siRNA transfection reagent (jetPRIME® transfection reagent, Illkirch, France) according to the manufacturer's instructions. The following siRNA oligonucleotides were purchased from GenePharma (Shanghai, China):

*DDR1*-siRNA#1: GAAUGUCGCUUCCGGCGUGUU

*DDR1*-siRNA#2: GAGCGUCUGUCUGCGGGUAUU

Knockout of *DDR1*. NSCLC cells were transduced with a CRISPR/Cas9 gene editing plasmid. NSCLC cells were first infected with Cas9-expressing lentivirus (Lenti-Cas9-puro, Genechem, Shanghai, China). Subsequently, NSCLC-Cas9 cells were infected with lentivirus targeting human DDR1 (lentiviral vectors purchased from Genechem). The following sgRNA sequences were used:

*DDR1*-sgRNA: AGTAACGCAGCCGGTAGCTC

Overexpression of wild-type DDR1 and kinase-dead mutant DDR1 K618A. Plasmid and primer design were carried out using SnapGene. Plasmid transfection was performed using PolyJet^TM^ reagent (SL100688, SignaGen, Rockville, MD, USA) according to the manufacturer's recommendations. 12-18 hours post-transfection, the cells were replenished with fresh complete medium and incubated for 36 hours before subsequent analysis.

Knockdown of *GLUT1*. NSCLC cells were transfected with siRNA oligonucleotides using siRNA transfection reagent (jetPRIME® transfection reagent, Illkirch, France) following the manufacturer's protocol. The following siRNA oligonucleotides were obtained from GenePharma (Shanghai, China):

*GLUT1*-siRNA#1: CAAAGUUCCUGAGACUAAAGG

*GLUT1*-siRNA#2: CCACGAGCAUCUUCGAGAAGG

*GLUT1*-siRNA#3: GAGGAGUUCUACAACCAGACA

Overexpression of GLUT1. Plasmid and primer design were carried out using SnapGene. Plasmid transfection was performed using PolyJet^TM^ reagent (SL100688, SignaGen, Rockville, MD, USA) according to the manufacturer's recommendations. 6 hours post-transfection, the cells were replenished with fresh complete medium and incubated for 16 hours before subsequent analysis.

### Animal studies

The sgRNA/Cas9 complex vector (Gemma Genetics, Hangzhou, China) was intratumorally injected into the transplanted tumor when it reached a volume of approximately 100 mm^3^. The composite vector was diluted with precooled sterilized PBS to achieve a final concentration of 1×10⁷ TU/50 μL. 50 μL of *DDR1* or control sgRNA/Cas9 composite virus was administered via intratumoral injection. The injections were administered every 2 days, for a total of 2 times. The volume of the transplanted tumor was calculated using the following formula: volume (mm^3^) = 0.5 × long diameter × short diameter^2^.

Relative tumor volume (RTV) = V_t_/V_0_, where V_0_ is the volume at the time of randomization. The tumor growth inhibition rate TGI (%) = (1 - V_treated_/V_control_) * 100, where V_treated_ and V_control_ are means of tumor volumes in the treated group and the control group.

Drug administration. The mice were divided into a control group and several experimental groups, including the docetaxel, anlotinib, and combination groups. Anlotinib was dissolved in 0.5% Carboxymethyl cellulose sodium (CMC-Na) to prepare the stock solution. Docetaxel was dissolved in the solvent specified in the instructions and stored accordingly. Mice were treated with indicated drugs, while those in the control group received an equivalent volume of 0.5% CMC-Na. For the anlotinib group: 0.8 mg/kg (for the A549 and PDX models) or 0.6 mg/kg (the PC9 model) anlotinib was administered daily for 14 days, followed by a 7-day interval, constituting one cycle (A549 model) or two cycles (PDX). The dose was selected based on dose conversion from human dosing regimens. For the *DDR1*-KO A549 xenograft model, the dosage of anlotinib was adjusted to 0.5 mg/kg daily for 14 days. For the other groups, the treatments were as follows. Docetaxel group: docetaxel was administered via intraperitoneal injection at a dose of 10 mg/kg on Day 1 (for the A549 model), or 5 mg/kg on Day 1 and Day 8 (for *DDR1*-KO A549 xenograft mice) or 15 mg/kg on day 1 (for the PC9 model). Combination group: the two drugs were administered in combination, following the respective dosing regimens described above.

### Bioinformatics analysis

DDR1 expression analysis was performed using the Xiantao Academic website (https://www.xiantaozi.com/). RNA-seq data from the TCGA database (https://portal.gdc.cancer.gov) were obtained from the STAR-alignd TCGA-LUAD and TCGA-LUSC cohorts, quantified as TPM value, and paired tumor and adjacent normal samples were selected. The statistical analysis was performed using a paired samples t-test.

Kaplan-Meier survival analysis was performed using the KM Plotter (https://kmplot.com). DDR1 mRNA expression data and corresponding survival data for NSCLC patients were downloaded from the following datasets: GSE102287, GSE14814, GSE157011, GSE19188, GSE29013, GSE30219, GSE31210, GSE3141, GSE31908, GSE37745, GSE4573, GSE50081, GSE68465, as well as from the TCGA database. Cutoff values between the lower and upper quartiles of expression were calculated, and the optimal cutoff with the most significant split was selected as the threshold 23.

### Immunohistochemistry

FFPE slides were dewaxed with xylene, rehydrated through graded ethanol, heated in citric acid (pH 5.65) using a microwave oven for antigen repair and blocked with 5% bovine serum albumin (BSA) solution. Subsequently, primary antibodies were applied, followed by staining with secondary antibodies. To semi-quantitatively assess the p-DDR1 immunostaining results, evaluable sections were classified into four IHC scores according to the percentage of p-DDR1-positive cells among the total number of tumor cells: IHC score 0, 0% positive; IHC score 1, 1-10% positive; IHC score 2, 11-50% positive; and IHC score 3, >50% positive. The association between PFS and the level of p-DDR1 in NSCLC patients was calculated using the Pearson correlation test.

Antibodies. Rabbit polyclonal anti-DDR1 (Huabio, HA500141); rabbit polyclonal anti-phosphorylated DDR1 (p-DDR1) (Invitrogen, PA5-37501); rabbit monoclonal anti-Ki67 (CST, 12202). HRP conjugated alpaca anti-rabbit IgG (Huabio, HA1031) was used as the secondary antibody.

### Western blotting

Tissues and cell samples were homogenized in lysis buffer containing 0.3% (v/v) NP40, 0.3% (v/v) Triton X-100, 0.25 μg/mL leupeptin, 0.1% PMSF and 0.1% Na_3_VO_4_. Protein lysates were then separated by sodium dodecyl sulfate-polyacrylamide gel electrophoresis (SDS-PAGE) using 8%, 10% or 12% gels, with 20-30 μg of total protein loaded per sample. Subsequently, the gels were transferred to polyvinylidene fluoride (PVDF) membranes (Merck Millipore, USA) using a wet transfer system. Next, the membranes were incubated with the corresponding primary and secondary antibodies and the signals were detected using Western Lightning Plus-ECL substrate (NCM Biotech, Suzhou, China). Band intensities were quantified using ImageJ software. Regions of interest (ROIs) were drawn around each band with identical dimensions for all lanes. Background subtraction was performed using the rolling ball method (radius = 50 pixels). Values were normalized to β-actin loading controls run on the same gel. Normalized values were expressed as relative fold change compared to the control group. Data are representative of three independent experiments.

Antibodies: The following primary antibodies were used: rabbit polyclonal anti-DDR1 (Huabio, HA500141); rabbit polyclonal anti-p-DDR1 (Invitrogen, PA5-37501); rabbit polyclonal anti-c-PARP (Abcam, ab4830); rabbit polyclonal anti-GLUT1(CST, 5625); rabbit monoclonal anti-PDK1 (Huabio, ET1704-66); rabbit monoclonal anti-HK2 (Abcam, EPR20839); rabbit monoclonal anti-β-ACTIN (Huabio, SY25-03). HRP conjugated alpaca anti-rabbit IgG (Huabio, HA1031) was used as the secondary antibody.

### Cell viability assay

Cell viability was assessed via the sulforhodamine B (SRB) or cell counting kit 8 (CCK8) colorimetric assay. The absorbance was measured using a microplate reader (Thermo Electronics Marietta, OH) at the respective wavelengths (515 nm for SRB and 450 nm for CCK8). Each cell viability assay was performed in three independent experiments, each in triplicate.

### Kinase profiling analysis

A total of 207 kinase inhibition assays targeting the human kinome were performed by ICE Bioscience (Hangzhou, China). *In vitro* kinase panel profiling was performed using the homogeneous time-resolved fluorescence (HTRF)-based and ADP-Glo kinase assays. A phylogenetic tree was generated using the KinMap available at https://www.kinhub.org.

### Synergy index (SI) calculation

The SI of the combination of docetaxel and anlotinib was calculated via the Bliss independence principle 24. At various concentrations of docetaxel and 150 nM anlotinib, the expected inhibition rate (E_expected_) was calculated using the following equation. E represents the inhibition rate obtained from the cell viability assay results, where A represents anlotinib and D represents docetaxel. The difference between observed inhibition rate (E_observed_) and the E_expected_ at the same combination dose is defined as the synergy index (SI), which is interpreted as follows: SI > 0 indicates synergy, SI = 0 indicates independence, SI < 0 indicates antagonism. Each experiment was performed at least three times in triplicate.

E_expected_ = E_A_ + E_D_ - E_A_ × E_D_

SI = E_observed_ - E_expected_

### Flow cytometry analysis

For Annexin V/propidium iodide (PI) staining assay, cells were seeded into 6-well plates at a density of 2 × 10^5^ cells per well for A549 cells and 3 × 10^5^ cells per well for PC9 cells, followed by incubation for 24 hours. Subsequently, the cells were treated with the indicated drugs for 48 hours. At the end of the incubation period, the cells were harvested and washed with PBS. The FITC Annexin V Apoptosis Detection Kit I (556547, BD Biosciences, San Jose, CA, USA) was utilized to detect the apoptotic rate using a CytoFLEX cytometer (B53015, Beckman Coulter, Brea, USA) according to the manufacturer's instructions. Total apoptosis = Early apoptosis (Annexin V⁺/PI⁻) + Late apoptosis/necrosis (Annexin V⁺/PI⁺). Data represent three independent experiments, each performed in triplicate.

### TdT-mediated dUTP nick end labeling (TUNEL) assay

A one-step TUNEL apoptosis assay kit (C1088, Beyotime, Shanghai, China) was used to detect cell apoptosis according to the manufacturer's instructions. Briefly, tissue sections were dewaxed and rehydrated, followed by pretreatment with Proteinase K (ST532, Beyotime, Shanghai, China) working solution. Subsequently, TUNEL detection solution was added to the slides, which were incubated with the tissue samples for 60 minutes at 37 °C in a humidified chamber protected from light. Finally, the nuclei were stained with 4′, 6-diamidino-2-phenylindole (DAPI) (D212, Dojindo, Kumamoto, Japan) and the sections were mounted for observation under a fluorescence microscope.

### Glycolytic activity assay

Seahorse XFe96 Extracellular Flux Analyzer (Seahorse Bioscience, North Billerica, MA, USA) was utilized to evaluate the glycolytic activity. A549 or A549 *DDR1* KO cells were seeded into XF96 multiwell plates and incubated at 37 °C with 5% CO₂ for 5 hours. Subsequently, the cells were treated with the indicated drugs before the assay was conducted. One hour before measurement of glycolytic activity, the culture medium was replaced with phenol red-free minimal DMEM (0 mM glucose), supplemented with 143 mM NaCl, 2 mM glutamine, and 1 mM sodium pyruvate, and adjusted to pH 7.4. The extracellular acidification rate (ECAR) was then recorded under basal conditions.

### Lactate production assay

A549 or A549 *DDR1* KO cells were treated with the indicated drugs for 48 h. The lactate in the culture medium was measured using the Lactate Assay Kit (BioVision, Milpitas, CA, USA) according to the manufacturer's instructions. The concentration of lactate was determined via the lactate standard curve.

### Proteomic profiling

Protein samples were extracted from the xenograft tumors of nude mice treated with the indicated drugs. The iTRAQ-based proteomic analysis was then conducted by APTBIO (Shanghai, China). Protein identification and quantification were performed using ProteinPilot software, and differentially expressed proteins were identified with a fold change > 1.2 and p-value < 0.05.

### Clinical trial design and sample size estimation

A single-arm, two-center, prospective phase Ⅱ clinical trial was conducted, enrolling advanced NSCLC patients who had previously failed with platinum-based chemotherapy. This trial was conducted from December 2018 to February 2023. A total of 45 patients signed informed consent form. The clinical trial was approved by the Ethics Committee of Hangzhou First People's Hospital (Medical Scientific Research Ethics No.2018-12-01 and 2020-45-01) and was registered with chictr.org.cn, number ChiCTR1800020011.

For this study, randomization or blinding was not applicable. All participants with advanced NSCLC received docetaxel at a dosage of 60 mg/m^2^ for four cycles, along with anlotinib at a dosage of 12 mg orally per day for 2 weeks every 3 weeks, continuing until disease progression, intolerant toxicity, or withdrawal of consent. Dose reduction of anlotinib from 12 mg to 10 mg daily was permitted for patients experiencing intolerable toxicity. Tumor response was assessed via CT scans with RECIST 1.1 every 6 weeks until disease progression. Participants were also followed up until they were lost to follow-up or deceased. The primary endpoint was ORR, while the secondary endpoints included disease control rate (DCR), PFS, OS and safety.

Previous literature has reported that the ORR for docetaxel monotherapy is approximately 9% 7. It was hypothesized that the combination of anlotinib with docetaxel would increase the ORR to 25%. To test this hypothesis, one-sided significance level (α) of 0.05 and a desired test power of 0.8 were set. Based on these parameters, the required sample size was calculated to be 35 cases. However, considering an anticipated dropout rate of 20%, 44 patients were planned for enrollment. Ultimately, 45 patients were successfully recruited and participated in this study.

### Statistical analysis

Statistical analyses were conducted using the GraphPad Prism 9 (RRID:SCR_002798). Each experiment was performed in triplicate and the results are expressed as the mean ± standard deviation (SD), unless otherwise stated. For comparisons between two groups, an unpaired two-tailed Student's t-test was used. Differences between more than 2 groups with a single variable were assessed using one-way ANOVA. Two-way ANOVA was used for comparisons involving two variables across two or more groups. Specific statistical analyses used for each figure are detailed in the figure legends. The symbols used to indicate significance levels are as follows: ns indicates no significance; *, *p* < 0.05; **, *p* < 0.01; ***, *p* < 0.001; and ****, *p* < 0.0001. Survival was estimated using the Kaplan-Meier method.

## Results

### DDR1 expression is negatively correlated with outcome and contributes to the low sensitivity to docetaxel in NSCLC

Several previous studies have suggested that the DDR1 expression can be induced by treatment and is involved in the resistance to chemotherapy in various cancers 11,14,25,26. To elucidate the potential associations between DDR1 expression and tumorigenesis and prognosis of NSCLC, we leveraged publicly available data from The Cancer Genome Atlas (TCGA) and Gene Expression Omnibus (GEO) databases. Specifically, analysis of mRNA-sequencing data from 107 paired primary NSCLC tumors and adjacent normal tissues revealed that DDR1 expression was significantly higher in tumor tissue than in matched noncancerous tissue (*p* < 0.0001) (Fig. 1A). Furthermore, to comprehensively evaluate the impact of DDR1 expression on prognosis, we integrated the TCGA and GEO mRNA sequencing databases of 2166 patients with NSCLC, using an automatically optimal cutoff value 23. We found that high DDR1 expression was also significantly related to worse OS. Specifically, patients with high DDR1 expression presented an 18% increased risk of death, compared with those with low DDR1 expression (hazard ratio = 1.18, *p* = 0.0081) (Fig. 1B).

DDR1 kinases mediate cellular function in their phosphorylated form (p-DDR1). To elucidate the potential relationship between p-DDR1 levels and the sensitivity to docetaxel in NSCLC, FFPE samples from 12 patients with advanced NSCLC who failed previous platinum-based chemotherapy were subjected to an IHC assay, with baseline characteristics shown in Fig. S1A. The results revealed that the patients with lower levels of DDR1 phosphorylation had prolonged PFS from 125 to 428 days, whereas those with higher levels of DDR1 phosphorylation had short PFS from 27 to 68 days (Fig. 1C and D). Correlation analysis further revealed that the level of p-DDR1 was significantly negatively correlated with PFS (Fig. S1B). In addition, docetaxel increased the p-DDR1 level in NSCLC cells including *KRAS* mutated A549, PC9 harboring *EGFR* exon 19 deletion and H1299 harboring *TP53* deletion, while cell apoptosis was induced with increased cleaved poly (ADP-ribose) polymerase (c-PARP) protein expression in all above three cell lines in a concentration- and time-dependent manner (Fig. 1E; Fig. S1C-F).

The results of the CCK8 assay and optical microscopy revealed that silencing *DDR1* could increase the inhibitory efficacy of docetaxel (Fig. 1F and G). Similarly, silencing DDR1 aggravated docetaxel-induced cell apoptosis (Fig. 1H). We further deleted *DDR1* expression in A549 cells via CRISPR/Cas9, and the cell survival curve revealed a leftward shift in the IC_50_ value of docetaxel from 23 nM to 9 nM (Fig. 1I). We further deleted *DDR1* expression in A549 cell-derived xenografts via CRISPR/Cas9 (Fig. 1J), and the deletion efficacy was verified via an IHC assay (Fig. 1K). Consistent with the *in vitro* findings, the combination treatment group exhibited significantly smaller tumor volumes compared to monotherapy groups (Fig. 1L). Taken together, these data demonstrated that DDR1 was negatively correlated with the outcome and sensitivity to docetaxel in NSCLC, and that the deletion of *DDR1* could enhance the anti-tumor effects of docetaxel.

### Anlotinib enhances sensitivity to docetaxel by inhibiting DDR1 *in vitro*

To enhance the anti-tumor effects of docetaxel through pharmacological approaches, we aimed to identify clinically available DDR1 inhibitors. Previous studies have suggested that anlotinib may be a potential DDR1 inhibitor 19,27, which needs to be further confirmed. We utilized the kinase profiling platform from ICE bioscience to evaluate the inhibitory effect of anlotinib across a panel of 207 kinases. As shown in Fig. 2A, at a concentration of 1 μM, anlotinib significantly inhibited a broad spectrum of tyrosine kinases. Notably, anlotinib exhibited potent inhibitory activity against DDR1 (Fig. 2B). Western blotting (WB) verified that anlotinib could significantly down-regulate the level of phosphorylated DDR1 in a time- and concentration- dependent manner in A549, PC9 and H1299 cells (Fig. 2C; Fig. S2A and B), indicating a potential combined effect between anlotinib and docetaxel. In line with our hypothesis, anlotinib significantly attenuated docetaxel-induced up-regulation of p-DDR1 and concomitantly triggered a pronounced expression of c-PARP (Fig. 2D and E; Fig. S2C). Flow cytometry further confirmed that the combination of anlotinib and docetaxel significantly promoted cell apoptosis compared with either treatment alone (Fig. 2F and G; Fig. S2D). Consistent with these findings, optical imaging revealed that co-treatment with anlotinib and docetaxel markedly increased cell suspension and death, and the cell survival curve exhibited a leftward shift with a 2- to 3- fold decrease in the IC_50_ value of docetaxel in all three cell lines (Fig. 2H and I; Fig. S2E and F). To quantify the combined efficacy of anlotinib with docetaxel in NSCLC cells, we calculated the synergy index (SI) according to the Bliss independence criterion 22,24. As depicted in Fig. 2J, co-treatment yielded synergistic cytotoxicity across A549, PC9, and H1299 cells, achieving SI values significantly beyond the additivity threshold (SI>0.18). These data demonstrated that anlotinib could increase the sensitivity to docetaxel by suppressing DDR1 *in vitro*.

### Anlotinib enhances the anti-tumor efficacy of docetaxel by inhibiting DDR1 in NSCLC cell-derived and patient-derived tumor xenografts

Next, we employed subcutaneous xenograft models derived from A549 and PC9 cells, as well as patient-derived lung adenocarcinoma (LUAD), to evaluate whether anlotinib could enhance the efficacy of chemotherapy *in vivo*. As described in the methods section, the mice were intraperitoneally injected with docetaxel or/and orally administered with anlotinib and then euthanized (Fig. S3A and B). Compared to monotherapy, the concomitant administration of anlotinib and docetaxel produced significant suppression of the tumor volume and weight in both A549 (Fig. 3A-C) and PC9 xenografts (Fig. 3D-F).

We next validated these findings in a previously established patient-derived xenograft (PDX) model of LUAD 22 (Fig. S3C). Before the second cycle of administration, the mice in the control group were euthanized ahead of schedule out of humanitarian concern because of the size of tumors (Fig. 3G). Combined treatment with these two drugs markedly suppressed tumor growth. Notably, complete tumor regression was observed in three mice (Fig. 3H and I). Additionally, the Ki67 staining results revealed that the combined treatment inhibited the proliferation of cancer cells *in vivo* (Fig. 3J and K). TUNEL assays revealed that, compared with docetaxel alone, anlotinib alone or the control group, combined treatment induced a greater degree of apoptosis (Fig. 3L and M). IHC staining demonstrated that the combination treatment significantly attenuated the levels of p-DDR1 in tumors (Fig. 3N). Consistent with the observations in cell-line xenografts, IHC and TUNEL assays demonstrated that anlotinib-docetaxel combination markedly attenuated proliferative capacity (Ki67⁺ fraction), provoked apoptosis, and down-regulated p-DDR1 expression compared to either single agent regimen in the PDX model (Fig. 3O). To ensure the safe clinical application of the combination therapy, all the mice were weighed two to three times a week, and a range of serum enzymes associated with organ function including the liver, kidney and heart, were tested. No significant weight loss was observed in any treatment group (Fig. 3P; Fig. S3D and E). Serum chemistry analysis revealed no significant hepatic, renal, or cardiac toxicity across all treatment groups (Fig. S3F-H). Taken together, these findings indicate that anlotinib could enhance the anti-tumor activity of docetaxel *in vivo* without causing significant organ toxicity.

Moreover, to further investigate whether the combined effect of anlotinib and docetaxel is associated with DDR1 inhibition, *DDR1*-KO A549 xenograft mice were generated through two intratumoral injections of the sgRNA/Cas9 mixture (Fig. 4A). In *DDR1* WT A549 xenografts, the results were consistent with the previous data, showing that the combination of anlotinib and docetaxel significantly attenuated tumor growth compared with docetaxel alone or anlotinib alone (Fig. 4B-D). In contrast, anlotinib failed to enhance the anti-tumor activity of docetaxel in *DDR1*-KO A549 xenografts (Fig. 4E-G), the *DDR1* knockout efficiency of which was verified by an IHC assay (Fig. 4H). These analyses indicated that the DDR1 expression was related to the anti-tumor ability of docetaxel and anlotinib potentiated the anti-tumor efficacy of docetaxel by targeting DDR1.

### Anlotinib enhances the sensitivity to docetaxel by inhibiting the DDR1-mediated glycolysis pathway in lung cancer

To illuminate the underlying mechanism of the combined effect of anlotinib with docetaxel and the role of DDR1, we subsequently performed iTRAQ^TM^-labeling proteomics on xenograft tumor tissues and screened for reliable proteins against the protein library in UniProt. As illustrated in the Venn diagram, comparative proteomic analysis revealed that the expression levels of 92 proteins were concurrently downregulated in both the anlotinib group (relative to the control group) and the anlotinib-docetaxel combination group (relative to the docetaxel monotherapy group) (Fig. 5A). KEGG pathway enrichment analysis of these 92 proteins revealed that metabolism-related pathways were significantly enriched, with a particular emphasis on the glycolytic pathway (Fig. 5B).

DDR1 has been reported to be able to regulate metabolic reprogramming in breast cancer cells 28, indicating that the glycolytic pathway might be involved in the combined efficacy of anlotinib and docetaxel. We investigated the expression levels of key glycolytic enzymes by WB. Upon exposure to docetaxel, the expression levels of GLUT1, HK2 and PDK1 were upregulated in A549 cells, while there was an apparent downregulation in the combination treatment compared with docetaxel monotherapy (Fig. 5C; Fig. S4A). GLUT1 expression showed similar trends in other NSCLC cells, such as PC9 and H1299 cells (Fig. 5D; Fig. S4B and C). Additionally, docetaxel increased GLUT1 expression in a dose-dependent manner, whereas anlotinib inhibited GLUT1 expression in A549 cells (Fig. S4D). To explore the relationship between *DDR1* expression and glycolysis, *DDR1*-KO A549 cells were utilized. The results demonstrated that GLUT1 expression was significantly downregulated in DDR1-KO cells, and while reintroduction of wild-type DDR1 (DDR1-WT) fully restored GLUT1 levels, transfection with a kinase-dead DDR1 mutant (DDR1-KD) failed to rescue its expression (Fig. 5E). These findings indicate that DDR1 kinase activity is essential for glycolytic regulation, as glycolytic suppression is reversed by *DDR1*-WT but not by *DDR1*-KD. Furthermore, WB revealed that the level of GLUT1 and c-PARP were comparable in *DDR1*-KO cells treated with either docetaxel alone or in combination (Fig. 5F; Fig. S4E and F). To strengthen the attribution of drug synergy specifically to DDR1 inhibition, we performed rescue experiments in *DDR1*-KO cells using *DDR1*-WT and *DDR1*-KD. The results demonstrated that docetaxel-induced apoptosis was restored by *DDR1*-WT but not by* DDR1*-KD, confirming that DDR1 kinase activity is essential for the chemosensitization phenotype (Fig.5G).

To further investigate whether glycolysis is inhibited by anlotinib, we employed a Seahorse XF Analyzer to measure the levels of glycolysis, which revealed that *DDR1*-KO A549 cells exhibited pronounced reduction in both basal extracellular acidification rates (ECARs) and lactate levels relative to *DDR1*-WT A549 cells, indicating a suppression of glycolytic activity (Fig. 5H and I). As shown in Fig. 5J and K, the ECAR and lactate levels indicated that anlotinib attenuated the glycolytic activity, whereas docetaxel promoted it. As shown in Fig. S4G, under docetaxel treatment, *DDR1*-WT restored both ECAR and lactate levels to those comparable to *DDR1*-KO A549 cells, whereas *DDR1*-KD failed to rescue these glycolytic parameters, confirming that DDR1 kinase activity is essential for glycolytic regulation. Furthermore, in NSCLC cells with *GLUT1* knockdown, which mimics glycolysis inhibition, docetaxel exhibited a more significant inhibition of cell viability, as evidenced by a leftward shift in the cell survival curve and increased induction of apoptosis (Fig. 5L and M; Fig. S4H-J). To directly test whether glycolysis restoration reverses the sensitization phenotype, we performed *GLUT1* overexpression rescue experiments. As shown in Fig. 5N, *GLUT1* overexpression significantly attenuated combination-induced apoptosis, as demonstrated by reduced c-PARP levels compared to vector control. Collectively, our findings suggested that anlotinib potentiated the anti-tumor efficacy of docetaxel through inhibiting DDR1-mediated glycolysis pathway.

### Clinical efficacy and safety of the combination of anlotinib and docetaxel in advanced NSCLC patients with pre-treatment

A total of 48 patients signed the informed consent form, 3 patients were excluded, and 45 patients were ultimately enrolled for efficacy and safety analysis. At the time of data cutoff (February 1, 2023), the median follow-up duration was 14.0 (range, 2.7-49.3 months), 40 patients had passed away and all patients had discontinued treatment, with disease progression in 35 patients, intolerance to adverse events in 2 patients, and other reasons in 8 patients (Fig. 6A). The baseline characteristics of the 45 enrolled patients were summarized in Table S1. Among the 45 enrolled patients, the median age was 61 years (range, 37 to 74). Thirty-five (77.8%) patients were male, 43 (95.6%) patients were in stage IV and only 2 patients (4.4%) were in stage IIIB. Regarding performance status, 37 (82.8%) patients had an ECOG score of 1. In terms of histology, 30 (66.7%) patients had adenocarcinoma, 12 (26.7%) patients had squamous carcinoma and 3 (6.7%) patients had other histology. Regarding molecular status, 28 (62.2%) patients had no *EGFR* mutation or *ALK*/*ROS1* fusion, while 9 (20%) patients harbored *EGFR* mutation only or 3 (6.7%) patients had *ALK* fusions only. Prior therapies included targeted therapy in 14 (31.1%) patients, checkpoint inhibitors in 17 (37.8%) patients, and anti-VEGF antibody therapies in 14 (31.1%) patients.

Compared with historical data of docetaxel monotherapy 6, the combination therapy demonstrated a significantly higher ORR of 35.6% (vs. 4.2%) and disease control rate (DCR) of 84.4% (vs. 50.6%), whereas the incidence of stable disease (SD) was 48.9% (vs. 46.4%) and that of progress disease was only 6.67% (vs. 33.7%) (Fig. 6B). A waterfall plot of the response of 41 assessable patients was shown on Fig. 6E. The median PFS was 5.3 months (95% CI, 4.2-7.4), and median OS was 14.0 months (95% CI, 2.7-40.3) (Fig. 6C and D), both of which were notably longer than the respective 2.8 months and 9.6 months reported in the docetaxel arm of the Check-Mate 078 study 6. In one of the patients, the lung lesion exhibited a significant reduction in size, as illustrated in Fig. 6F. The most frequently reported treatment-related adverse events (TRAEs) included hand and foot skin reactions (28.9%, 13/45), hypertension (26.7%, 12/45), stomatitis (20.0%, 9/45), hemorrhage (20.0%, 9/45), leucopenia (17.8%, 8/45), Anaemia (15.6%, 7/45), thrombocytopenia (11.1%, 5/45) and electrolyte disturbances (11.1%, 5/45), with the majority of these events being grade 1 or 2 (Table S2). Overall, the combination of anlotinib with docetaxel was efficacious and tolerable in this clinical trial.

## Discussion

Our study reveals that DDR1 is intricately implicated in tumorigenesis and adverse prognosis of lung cancer, as well as in the diminished sensitivity to docetaxel. Docetaxel can up-regulate the level of DDR1 phosphorylation, which is instrumental in conferring low sensitivity to docetaxel. Concomitantly, our research demonstrates that anlotinib can increase the sensitivity to docetaxel by exerting an inhibitory effect on the glycolytic pathway via DDR1 suppression. To summarize, our study offers a promising strategy to increase the efficacy of docetaxel, thereby providing valuable insights for clinical decision-making in the management of NSCLC.

DDR1 activates multiple signaling pathways, including the MAPK, integrin, TGF-β, insulin receptor, and Notch pathways, all of which play critical roles in morphogenesis, proliferation, differentiation, adhesion, invasion, and metastasis across various solid tumors 10,29. In lung cancer, DDR1 phosphorylation was first reported to be elevated in a Cell study in 2007 30. Research has revealed that DDR1 inhibition suppresses tumor initiation and progression in KRAS-driven LUAD 11. Consistent with these findings, our bioinformatic analysis of public datasets further confirmed that high DDR1 expression was correlated with tumor occurrence in and poor prognosis of NSCLC. Notably, in *KRAS*- and *TP53*-mutant PDX models, combined inhibition of DDR1 and Notch signaling induced significant tumor regression, with efficacy comparable to that of cisplatin/paclitaxel chemotherapy 11. Additionally, TM4SF1, an oncogene implicated in chemoresistance, has been shown to modulate the DDR1/ERK/Akt-mTOR axis in NSCLC 31. Collectively, these findings underscore DDR1 as a promising therapeutic target to overcome resistance to anti-tumor agents.

Docetaxel is an essential chemotherapeutic agent in lung cancer, but its efficacy is still limited by resistance mediated by upstream EMT regulators such as ZEB1/2, TGF-β, miRNAs, and c-Myc 32. However, effective strategies to increase docetaxel sensitivity are still critically lacking. To date, only one previous study has reported that cystine supplementation can rebalance redox homeostasis and improve the sensitivity to docetaxel in A549/DTX cells 33. In our study, the high levels of DDR1 phosphorylation were associated with inferior PFS in NSCLC patients treated with docetaxel. Furthermore, the knockdown of *DDR1* significantly enhanced the anti-tumor efficacy of docetaxel across multiple models, thereby suggesting that DDR1 inhibitors could serve as a therapeutic strategy to overcome docetaxel insensitivity in NSCLC.

Several small-molecule DDR1 inhibitors, including sitravatinib, dasatinib, and nilotinib, as well as DDR1-targeting antibodies have been explored. However, dasatinib failed to demonstrate clinical benefit when added to chemotherapy for the treatment of advanced pancreatic cancer 34,35 or prostate cancer 36, leaving no preferred DDR1 inhibitor for clinical use. Our preliminary kinase profiling revealed that anlotinib is a potent DDR1 inhibitor. The combined anti-tumor activity of anlotinib and docetaxel was observed in NSCLC cell lines, cell-derived and patient-derived tumor xenografts, with favorable safety. Notably, complete tumor regression was achieved in three PDX models. In addition, our clinical trial further suggested that anlotinib significantly enhanced the efficacy of docetaxel compared with the LUME-Lung 1 study docetaxel plus nintedanib group 8, improving the ORR (35.6% vs. 4.4%), prolonging PFS (5.3m vs. 3.4m), and extending OS (14.0m vs. 10.1m). Overall, the combination of anlotinib with docetaxel demonstrated robust efficacy and tolerability across *in vitro*, *in vivo*, and clinical settings. Despite the promising clinical outcomes, several limitations of our clinical study should be acknowledged. First, this was a single-arm, open-label study without a randomized control group, which may introduce selection bias. Second, the sample size was relatively small, and all patients were only recruited from two centers, limiting the generalizability of the findings. Third, the study lacked a biomarker-driven stratification, and the optimal patient population that would most benefit from the combination therapy remains to be defined. Therefore, large-scale, multicenter, randomized controlled trials are warranted to validate our findings.

Energy metabolism reprogramming is a hallmark of cancer 37. To meet the demands of rapid proliferation, cancer cells rely primarily on glycolysis for energy production, a phenomenon known as the Warburg effect 38. This metabolic reprogramming, driven by activated oncogenes, mutated tumor suppressors, and the tumor microenvironment, not only enhances the aggressiveness of cancer cells but also contributes to drug resistance 39. In LUAD cells, tripartite motif-containing proteins enhance tumor cell proliferation, glycolysis, and cisplatin resistance by upregulating p-AKT levels 40. Key metabolic enzymes such as pyruvate kinase 2 (PKM2) and pyruvate dehydrogenase kinase 1 (PDK1) are crucial for tumor glucose metabolism. Notably, inhibiting PKM2/PDK1 can restore gefitinib sensitivity in EGFR-mutated NSCLC cells by reprogramming glucose metabolism 41. Similarly, in the colorectal carcinoma LoVo cell line, DDR1 promotes proliferation by activating the PI3K/AKT/PKM2 axis to enhance pyruvate kinase activity 42. DDR1 activation is known to stimulate the PI3K/AKT signaling pathway, in which HIF-1α serves as a key mediator of glucose metabolism regulation 43. Our proteomic data demonstrated significant downregulation of HIF signaling pathway-related factors following anlotinib plus docetaxel combination treatment, suggesting that the combination regimen may modulate DDR1-regulated glycolysis through the PI3K/HIF signaling pathway. In addition, DDR1 also drives aerobic glycolysis by upregulating glucose transporters and glycolytic enzymes via the p38 MAPK/c-Myc pathway in breast cancer, which accelerates tumor growth 44. In pancreatic cancer, DDR1 inhibition may suppress NF-κB-driven metabolic gene expression, thereby affecting mitochondrial metabolism 45. Here, we demonstrated that anlotinib could enhance the sensitivity to docetaxel by inhibiting DDR1-mediated glycolysis in NSCLC. These findings suggest that targeting energy metabolism reprogramming may represent a promising therapeutic strategy to improve docetaxel efficacy. However, given the complexity of metabolic networks and potential crosstalk with other signaling pathways (e.g., mTORC1, NF-κB), the precise molecular mechanisms linking DDR1 signaling to glycolytic reprogramming remain to be fully elucidated.

In conclusion, our study demonstrates that DDR1 expression is linked to lung cancer occurrence, drug resistance and poor clinical outcome. Anlotinib enhances the efficacy of docetaxel by inhibiting DDR1 phosphorylation and suppressing the glycolysis signaling pathway. Our findings not only highlight the new function of DDR1 in energy metabolism and drug resistance but also provide a preferable therapeutic strategy for clinical application.

## Supplementary Material

Supplementary figures and tables.

## Figures and Tables

**Figure 1:**
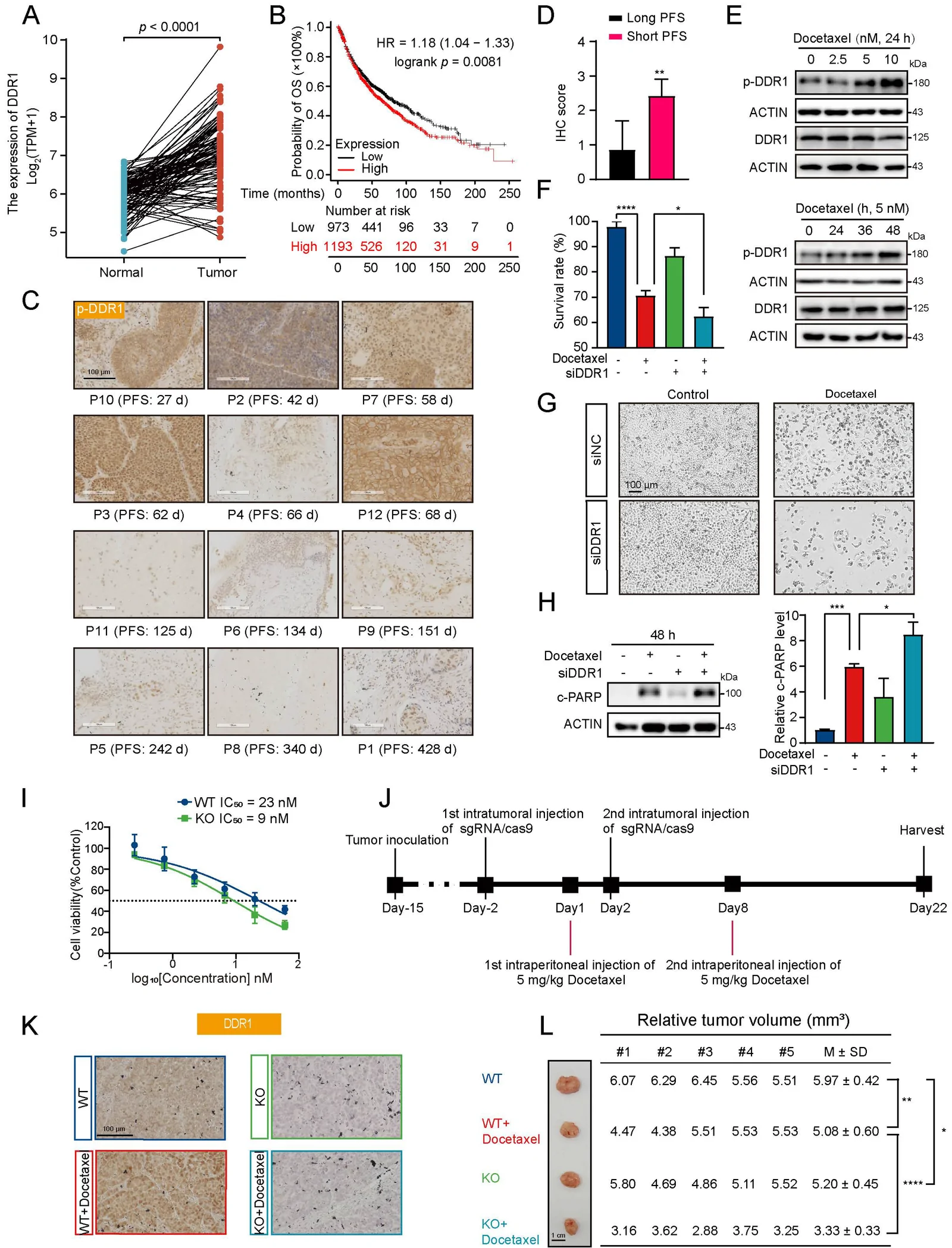
DDR1 expression is negatively correlated with outcome and contributes to the low sensitivity of NSCLC to docetaxel.** A** The mRNA levels of DDR1 between tumor tissue and adjacent normal tissue from TCGA database. **B** Overall survival for NSCLC patients from TCGA and GEO databases with high or low levels of DDR1 expression. **C** Representative images of p-DDR1 IHC in 12 NSCLC patients prior to docetaxel. Original magnification, 200×; scale bars, 100 μm.** D** IHC quantification of p-DDR1 with 3 regions of tumor tissue. Long-PFS (n = 6) and Short-PFS (n = 6) groups were stratified on a 90-day PFS threshold. Unpaired t test was used. **, *p* < 0.01. **E** The level of p-DDR1 expression in A549 cells under docetaxel exposure with various concentration for different time. See also Figure S1. **F** The cell survival rate of A549 cells with or without *DDR1* knockdown after treatment of 5 nM docetaxel (n = 3). The survival rate used ordinary one-way ANOVA with Tukey's multiple comparisons test. *, *p* < 0.05 and ****, *p* < 0.0001. **G** Cell morphology of A549 cells with or without *DDR1* knockdown after treatment of 5 nM docetaxel for 48 h using optical microscope observation. Original magnification, 100×; scale bars, 100 μm. **H** c-PARP protein level of A549 cells with or without* DDR1* knockdown treated with 5 nM docetaxel for 48 h and its quantification (n = 3). The data were analyzed by ordinary one-way ANOVA with Sidak's multiple comparisons test. *, *p* < 0.05 and ***, *p* < 0.001. **I** Cell viability of A549 cells with or without *DDR1* knockout following treatment of gradient-diluted docetaxel for 48 h (n = 3). **J** Animal experiment schematic diagram. **K** Representative images of DDR1 IHC in A549 cell-derived xenograft with or without DDR1 knockout. Original magnification, 200×; scale bars, 100 μm. **L** Relative tumor volume on the day of xenograft euthanasia (n = 5). Scale bars, 1 cm. Data are presented as the means ± SD and analyzed by one-way ANOVA with Fisher's LSD multiple comparisons test. *, *p* < 0.05; **, *p* < 0.01 and ****, *p* < 0.0001. One-way analysis of variance (ANOVA) tests with multiple comparison were performed. Each bar represents the mean ± SD. TPM indicates transcripts per kilobase million; HR indicates hazard ratio; PFS indicates progress free survival; p-DDR1 indicates phosphorylated-DDR1; WT indicates wild type; KO indicates knockout.

**Figure 2:**
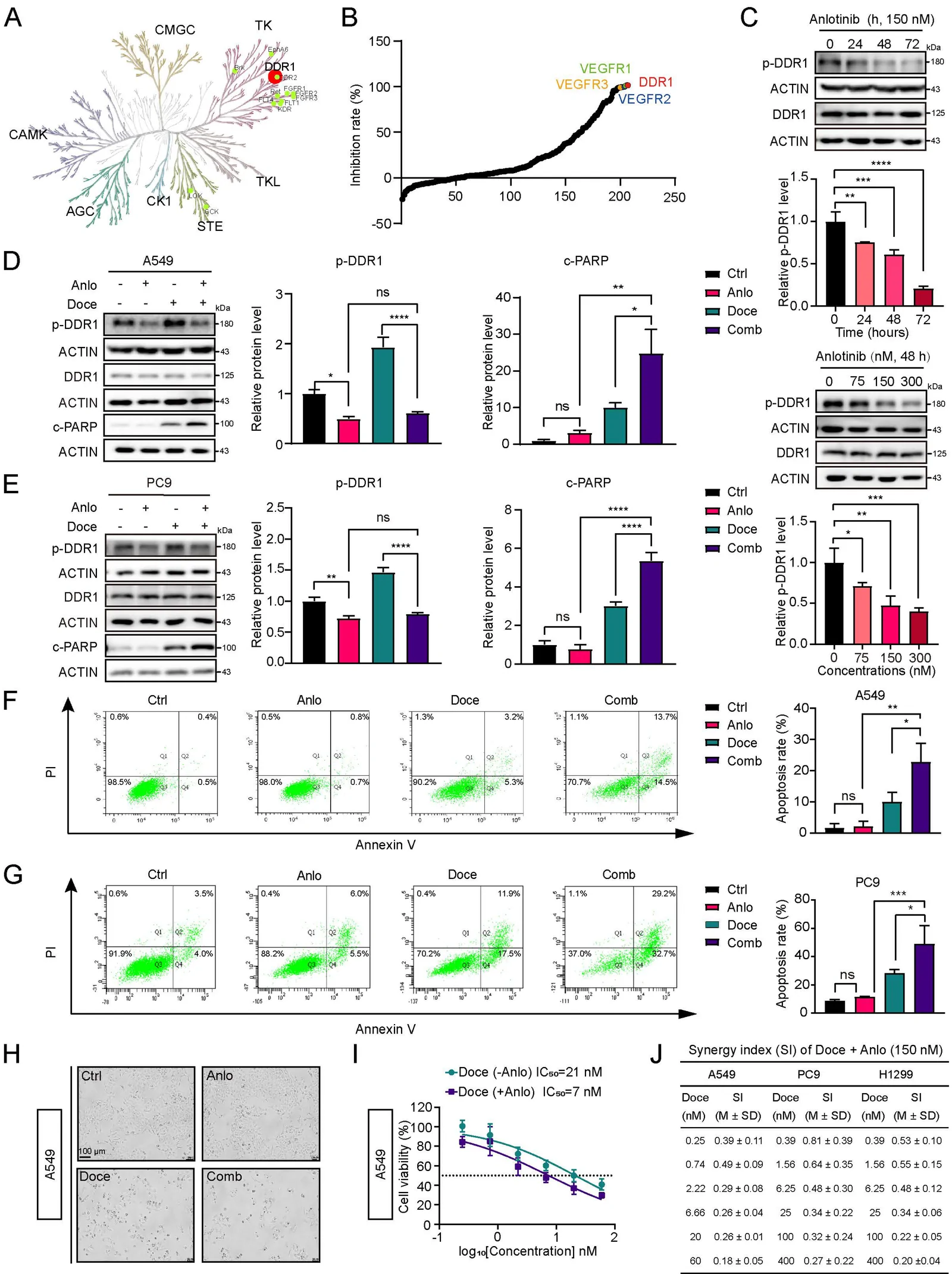
Anlotinib increases the sensitivity to docetaxel by inhibiting DDR1 *in vitro*. **A** Kinase profiling of 1 μM anlotinib with 207 kinases panel.** B** The DDR1 inhibition rate of 1 μM anlotinib. **C** Western blotting (WB) assay showed the level of p-DDR1 in A549 cells treated with various concentrations of anlotinib for different time points, and quantification of 3 independent experiments. The quantification of time- and concentration-dependent effect used ordinary one-way ANOVA with Dunnett's multiple comparisons test. *, *p* < 0.05; **, *p* < 0.01; ***, *p* < 0.001; and ****, *p* < 0.0001.** D** and** E** WB showed the levels of DDR1 phosphorylation (p-DDR1) and cleaved-PARP (c-PARP) in A549 cells (D) and PC9 cells (E) treated with 150 nM anlotinib and/or 5 nM docetaxel for 48 h, and quantification of 3 independent experiments. The data were analyzed by ordinary one-way ANOVA with Tukey's multiple comparisons test. ns indicates no significance; *, *p* < 0.05; **, *p* < 0.01; and ****, *p* < 0.0001.** F** and** G** Flow cytometry analyzed apoptosis with Annexin V-PI staining in A549 cells (F) and PC9 cells (G) treated with 150 nM anlotinib and/or 5 nM docetaxel for 48 h, and quantification of 3 independent experiments. The data were analyzed by ordinary one-way ANOVA with Tukey's multiple comparisons test. ns indicates no significance; *, *p* < 0.05; **,* p* < 0.01; and ***, *p* < 0.001.** H** Cell morphology was observed by optical microscopy in A549 cells treated with 150 nM anlotinib and/or 5 nM docetaxel for 48 h and cell viability of following treatment of gradient-diluted docetaxel and/or 150 nM anlotinib. Original magnification, 100×; scale bars, 100 μm. **I** IC50 values of docetaxel alone or in combination with anlotinib (48 h treatment) in A549 cells (n = 3).** J** Synergy index (SI) of A549 cells, PC9 cells and H1299 cells treated with 150 nM anlotinib and/or gradient-diluted docetaxel. Data are presented as the mean ± SD (n = 3).TK indicates tyrosine kinase; TKL indicates tyrosine-kinase-like family; STE indicates homologs of yeast sterile 7, sterile 11 and sterile 20 family; CK1 indicates casein kinase 1 family; AGC indicates the protein kinase family containing protein kinases A, G, and C; CAMK indicates calcium/calmodulin-dependent protein kinase; CMGC indicates cGMP-dependent protein kinases.

**Figure 3:**
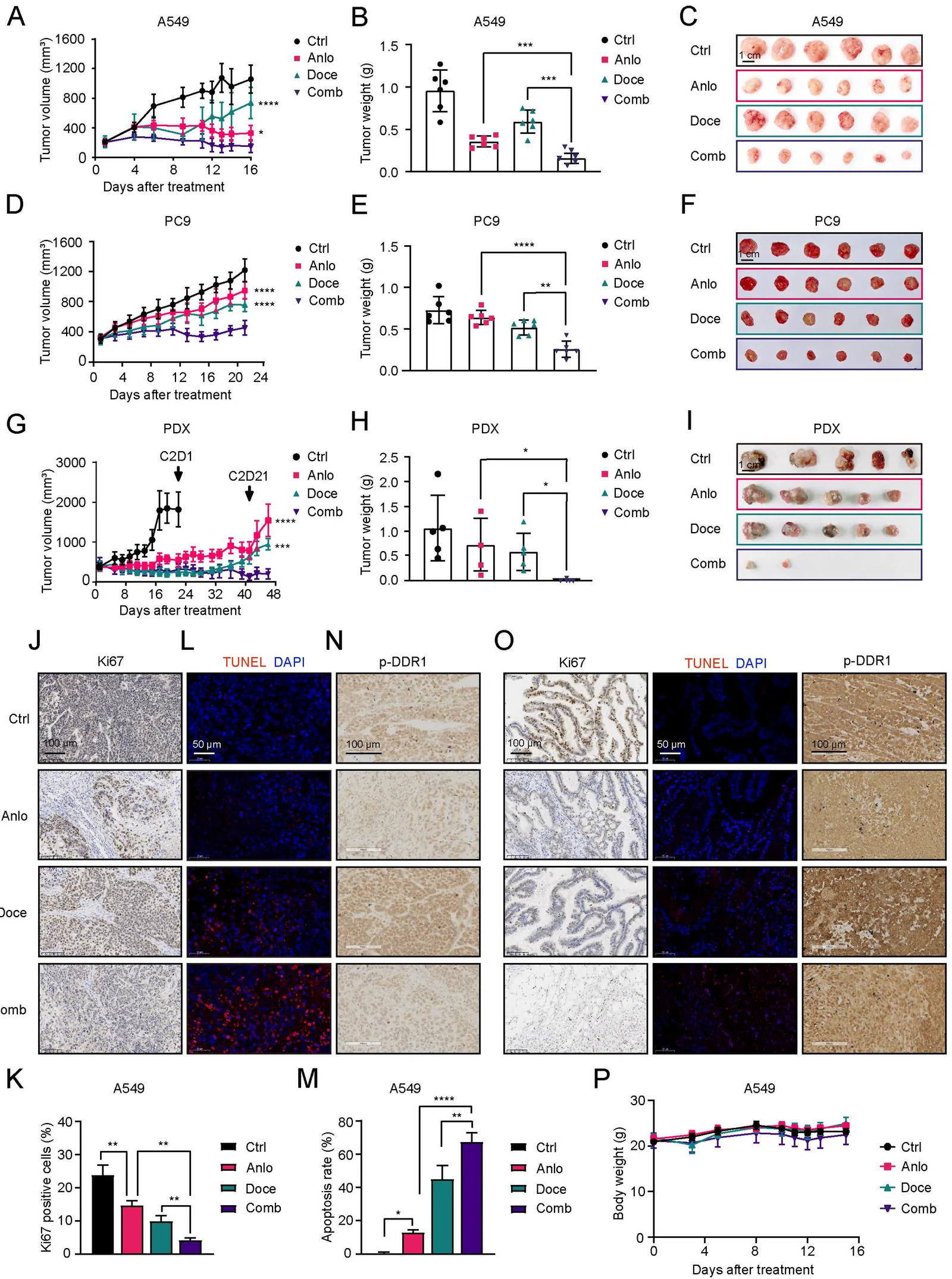
Combined anti-tumor activity of anlotinib and docetaxel in A549-derived, PC9 cell-derived and patient-derived tumor xenografts. **A** Tumor volume change curves for A549 cell subcutaneous xenograft-bearing mice after treatment with (2-hydroxypropyl)-β-cyclodextrin (in Ctrl group), anlotinib (0.8 mg/kg, daily gavage, from Day1 to Day14, in Anlo group), docetaxel (10 mg/kg, intraperitoneal injection, day1, in Doce group), and combined with the above two drugs (in Comb group), (n = 6). The quantification used two-way ANOVA with Tukey's multiple comparisons test. *,* p* < 0.05; ****, *p* < 0.0001. **B** Tumor weights on the day of A549 cell- derived xenograft euthanasia (n = 6). The data were analyzed by ordinary one-way ANOVA with Dunnett's multiple comparisons test. ***,* p* < 0.001. **C** Anatomical drawings of A549 cell-derived xenografts were taken after mice were euthanized. Scale bars, 1 cm.** D** Tumor volume change curves for PC9 cell subcutaneous xenograft-bearing mice after treatment with (2-hydroxypropyl)-β-cyclodextrin (in Ctrl group), anlotinib (0.6 mg/kg, daily gavage, from Day1 to Day14, in Anlo group), docetaxel (15 mg/kg, intraperitoneal injection, day1, in Doce group), and combined with the above two drugs (in Comb group), (n = 6). The quantification used two-way ANOVA with Tukey's multiple comparisons test. ****,* p* < 0.0001. **E** Tumor weights on the day of PC9 cell-derived xenograft euthanasia (n = 6). The data were analyzed by ordinary one-way ANOVA with Dunnett's multiple comparisons test. **, *p* < 0.01 and ****, *p* < 0.0001. **F** Anatomical drawings of PC9 cell-derived xenografts were taken after mice were euthanized. Scale bars, 1 cm. **G** Tumor volume change curves for PDX-bearing mice after treatment with (2-hydroxypropyl)-β-cyclodextrin (in Ctrl group), anlotinib (0.8 mg/kg, daily gavage for 14 days interval 7 days for two cycles, in Anlo group), docetaxel (10 mg/kg, intraperitoneal injection, day1, in Doce group), and combined with the above two drugs (in Comb group). The mice of control group were euthanized ahead of schedule because the tumor sizes were out of humanitarian concern (n = 5). The quantification used two-way ANOVA with Tukey's multiple comparisons test. ***, *p* < 0.001 and ****, *p* < 0.0001. **H** Tumor weights on the day of PDX euthanasia (n = 5). The data were analyzed by ordinary one-way ANOVA with Dunnett's multiple comparisons test. *,* p* < 0.05. **I** Anatomical drawings of PDX were taken after mice were euthanized. Scale bars, 1 cm. **J-N** Ki67 (J) and p-DDR1 (N) expression of the tumors in different groups of A549 cell xenograft model were determined using IHC. TUNEL assay detected the apoptosis in A549 cell xenograft (L). Quantification of Ki67 (K) and TUNEL (M) used ordinary one-way ANOVA with Tukey's multiple comparisons test. *, *p* < 0.05; **, *p* < 0.01 and ****, *p* < 0.0001. Scale bars, 50 or 100 μm. **O** Ki67 and p-DDR1 expression of the tumors in different PDX groups were determined by IHC. TUNEL assay detected the apoptosis in PDX. Scale bars, 50 or 100 μm. **P** The body weights of A549 xenograft-bearing mice were measured every two days in every group. Ctrl indicates control; Anlo indicates anlotinib; Doce indicates docetaxel; Comb indicates combination; C2D1 indicates the first day of the second cycle; C2D21 indicates the 21st day of the second cycle.

**Figure 4:**
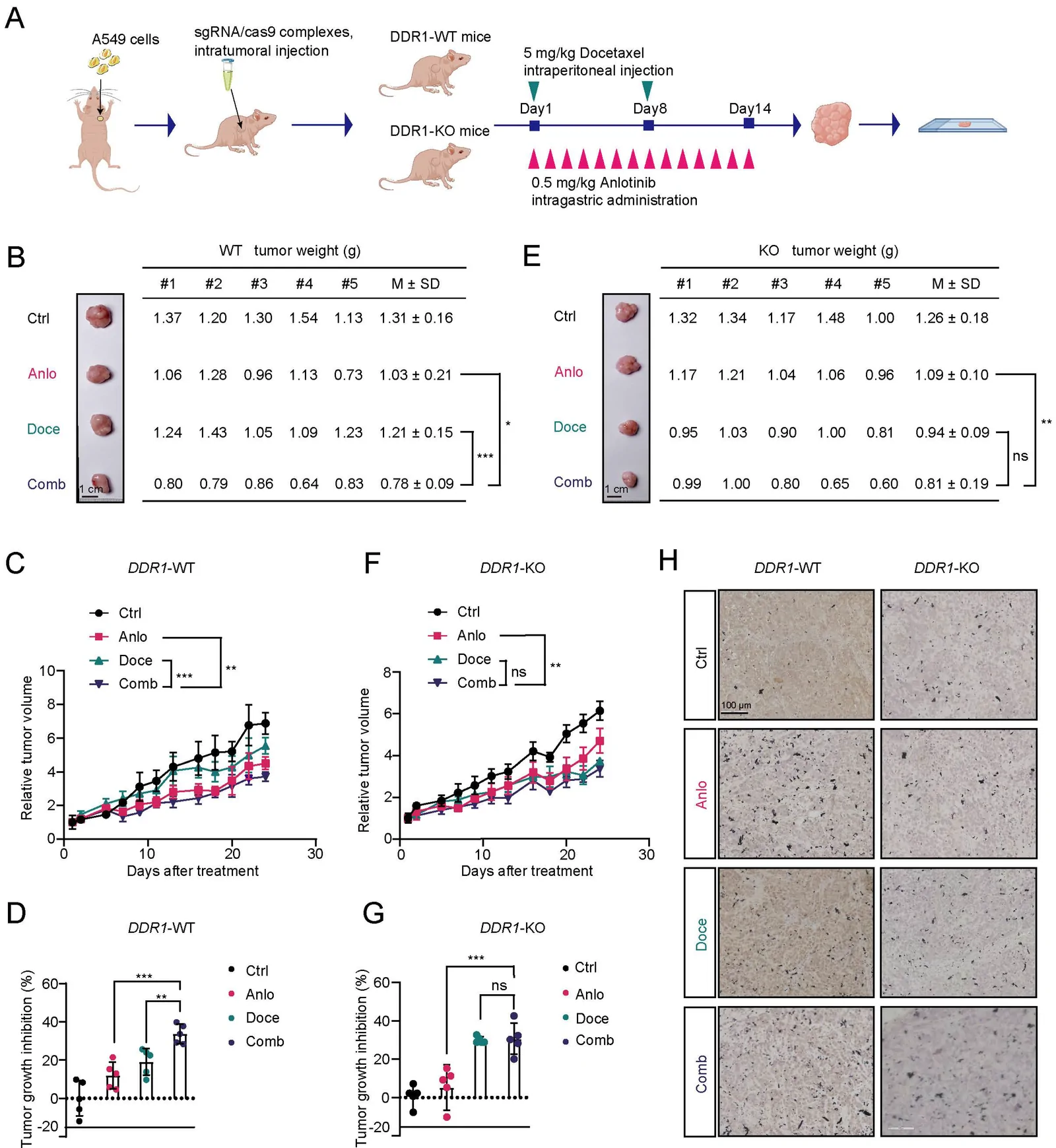
Anlotinib enhances the anti-tumor efficacy of docetaxel via inhibiting DDR1. **A** DDR1-knockout A549 cell-derived xenograft was constructed through CRISPR-Cas9 technology and the drug administration procedure is shown. **B** Tumor weights on the day of A549 xenograft euthanasia (n = 5). Scale bars, 1 cm. Data were presented as the mean ± SD and were analyzed by one-way ANOVA with Fisher's LSD multiple comparisons test. *, *p* < 0.05; and ***, *p* < 0.001. **C** and** D** Tumor volume change curves (C) for A549 cell derived xenograft (n = 5). Tumor growth inhibition (D) of A549 tumors on the day when mice were euthanized (n = 5). Tumor volume change curves were analyzed by two-way ANOVA with Fisher's LSD multiple comparisons test. Tumor growth inhibition was analyzed by one-way ANOVA with Fisher's LSD multiple comparisons test. **, *p* < 0.01; and ***, *p* < 0.001. **E** Tumor weights on the day of A549 *DDR1* knock-out xenograft euthanasia (n = 5). Scale bars, 1 cm. Data were presented as the mean ± SD and were analyzed by one-way ANOVA with Fisher's LSD multiple comparisons test. ns means no significance, **, *p* < 0.01. **F** and** G** Tumor volume change curves (F) for A549 cell with *DDR1-*knockout derived xenograft (n = 5). Tumor growth inhibition (G) of A549 tumors with *DDR1-*knockout on the day when mice were euthanized (n = 5). Tumor volume change curves were analyzed by two-way ANOVA with Fisher's LSD multiple comparisons test. Tumor growth inhibition was analyzed by one-way ANOVA with Fisher's LSD multiple comparisons test. ns indicates no significance; **, *p* < 0.001; and ***, *p* < 0.001. **H** DDR1 expression of various group xenografts detected by IHC. Scale bars, 100 μm. Ctrl indicates control; Anlo indicates anlotinib; Doce indicates docetaxel; Comb indicates combination; WT indicates wild type; KO indicates knock-out.

**Figure 5:**
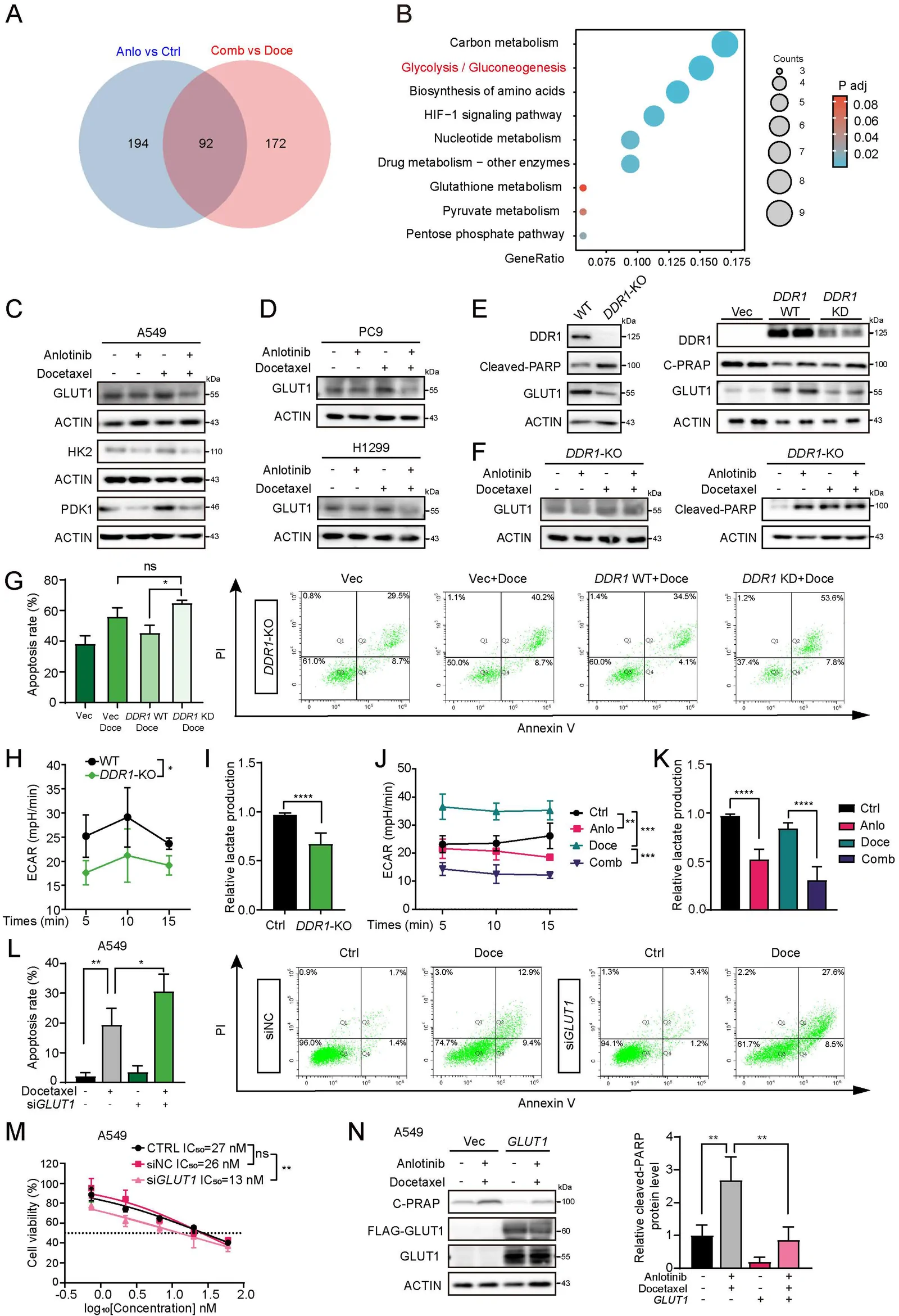
Anlotinib enhances the sensitivity to docetaxel by inhibiting the DDR1-mediated glycolysis pathway in lung cancer.** A** A Venn diagram revealed 92 significantly co-downregulated proteins in xenograft tumor tissues, identified through iTRAQ-labeled proteomics, in both the anlotinib monotherapy group (vs. control) and the combination therapy group (vs. docetaxel alone). **B** Bubble plot displayed that metabolism-related pathways were enriched by KEGG analysis. **C** WB evaluated the expression of key glycolytic enzymes including GLUT1, HK2 and PDK1 in A549 cells treated with 150 nM anlotinib or/and 5 nM docetaxel for 48 h. See also Figure S4A. **D** WB evaluated the expression of key glycolytic enzymes GLUT1 in PC9 cells and H1299 cells treated with 150 nM anlotinib or/and 5 nM docetaxel for 48 h. See also Figure. S4B and C. **E** The level of GLUT1 protein in A549 cells (WT), A549 KO cells (*DDR1*-KO) targeting *DDR1*, re-overexpression *DDR1* and *DDR1* K618A kinase-dead *(DDR1*-KD) mutant by WB (1 μg DDR1 overexpression plasmid was used in DDR1). **F** The level of GLUT1, c-PARP protein in *DDR1*-KO cells treated with 150 nM anlotinib or/and 5 nM docetaxel for 48h.** G** Apoptosis was analyzed by flow cytometry with Annexin V-PI staining in *DDR1*-KO A549 cells transfected with empty vector (Vec), wild-type *DDR1* (*DDR1*-WT), or kinase-dead *DDR1* K618A mutant (*DDR1*-KD) treated with or without 5 nM docetaxel for 48 h, and quantification of 3 independent experiments. The data were analyzed by ordinary one-way ANOVA with Tukey's multiple comparisons test. ns indicates no significance and *, *p* < 0.05. **H** and** I** ECAR and lactate production were measured in A549 cells with or without DDR1 knockout. The data were analyzed by ordinary two-way ANOVA with Sidak's multiple comparisons test (H) and Student's t-test (I). *, *p* < 0.05; ****, *p* < 0.0001. **J** and** K** Extracellular acidification rate (ECAR) and measurement of lactate production of A549 cells following 24 h treatment with 150 nM anlotinib and/or 5 nM docetaxel for 24 h. The data were analyzed by ordinary two-way ANOVA (J) and one-way ANOVA (K) with Tukey's multiple comparisons test (n = 3). **, *p* < 0.01; ***,* p* < 0.001; and ****, *p* < 0.0001. **L** Apoptosis was analyzed by flow cytometry with Annexin V-PI staining in A549 cells with or without *GLUT1* knockdown treated with 5 nM docetaxel for 48 h, and quantification of 3 independent experiments. The data were analyzed by ordinary one-way ANOVA with Tukey's multiple comparisons test. *, *p* < 0.05 and **, *p* < 0.01. **M** Dose-response curves of docetaxel for 48h upon A549 cells with or without *GLUT1* knockdown (siNC, si*GLUT1*), and blank control (CTRL). The data were analyzed by two-way ANOVA with Tukey's multiple comparisons test (n = 3). Error bars represent mean ± SD. ns indicates no significance and **, *p* < 0.01. **N** The level of GLUT1 protein in A549 cells transfected with empty vector (Vec), or *GLUT1* overexpression plasmid (*GLUT1*) treated with or without 150 nM anlotinib and 5 nM docetaxel for 48h by WB. The data were analyzed by ordinary one-way ANOVA with Tukey's multiple comparisons test (n = 3). Error bars represent mean ± SD. **, *p* < 0.01. Ctrl indicates control; Anlo indicates anlotinib; Doce indicates docetaxel; Comb indicates combination; WT indicates wild type; KO indicates knock-out; KD indicates kinase dead; CTRL indicates control; Vec indicates vector.

**Figure 6:**
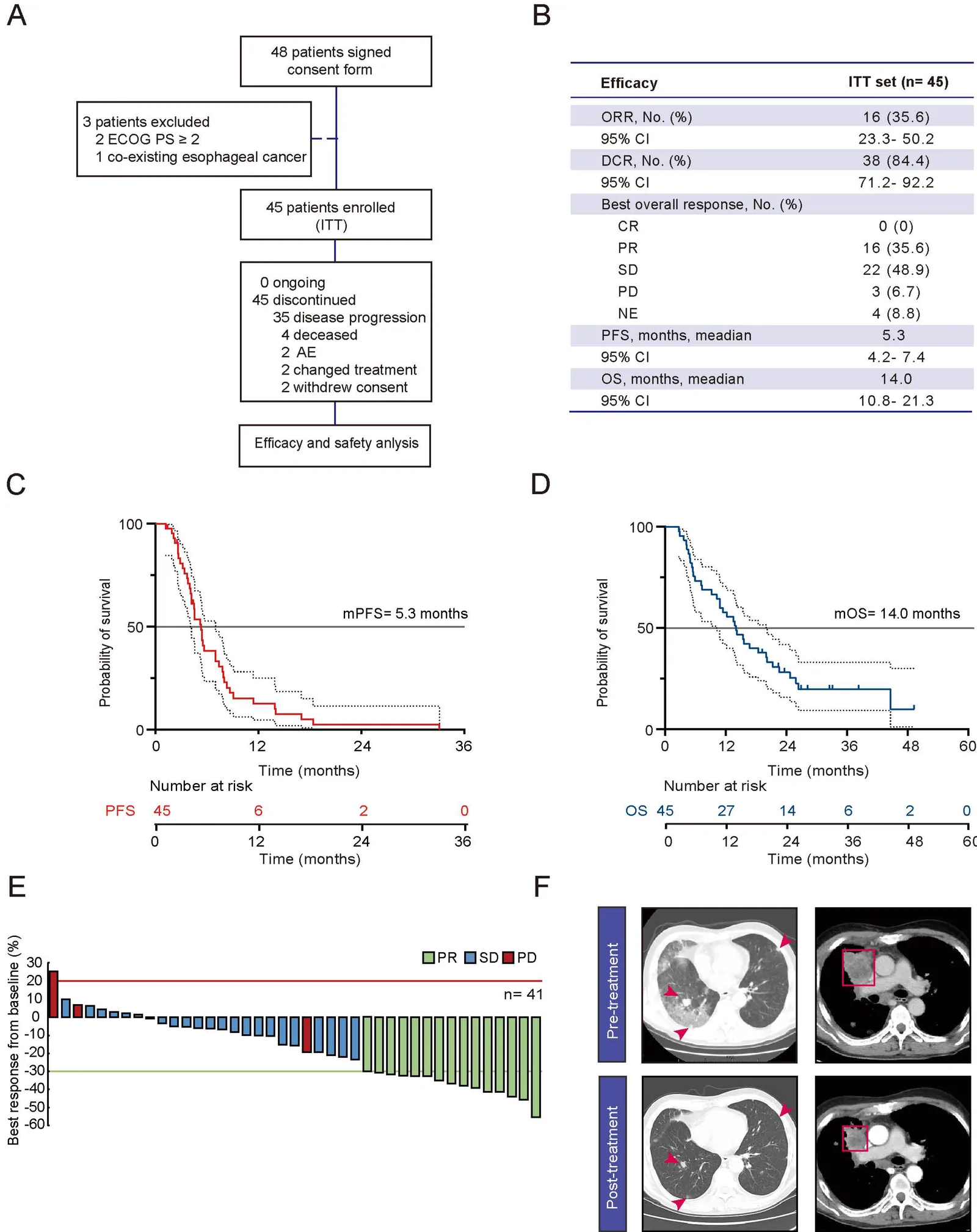
Clinical efficacy and toxicity of the combination of anlotinib and docetaxel in NSCLC patients with pre-treatment. **A** Flow diagram of this phase Ⅱ trial. **B** Summary of tumor response assessed by RECIST v1.1 in ITT population. **C.** Kaplan-Meier curve of progress-free survival (PFS) in the ITT population. **D** Kaplan-Meier curve of overall survival (OS) in the ITT population. **E** Waterfall plot of best percentage change in target lesion size in evaluable patients. **F** Representative CT images of a typical patient. ECOG indicates Eastern Cooperative Oncology Group, ITT indicates intention to treat, AE indicates adverse event, ORR indicates objective response rate, DCR indicates disease control rate, CR indicates complete response, PR indicates partial response, SD indicates stable disease, PD indicates progressive disease, mPFS indicates median progress-free survival, mOS indicates median overall survival.

## Data Availability

Data will be made available on request.

## Abbreviations

DDR1: Discoidin domain receptor 1
NSCLC: non-small cell lung cancer
KO: Knock out
ORR: overall response rate
PFS: progression-free survival
OS: overall survival
VEGFR: vascular endothelial growth factor receptor
FGFR: fibroblast growth factor receptor
RPMI-1640: Roswell park memorial institute 1640 medium
BALB/C: Bagg Albino C
NOD SCID: Non-Obese Diabetic-Severe Combined Immunodeficiency
IACUC: Institutional Animal Care and Use Committee
siRNA: Small Interfering RNA
GLUT1: Glucose Transporter 1
sgRNA/Cas9: Single-guide RNA/ CRISPR-associated protein 9
RTV: Relative tumor volume
TGI: Tumor growth inhibition rate
PDX: Patient-derived xenograft
TCGA: The Cancer Genome Atlas
LUAD: Lung adenocarcinoma
LUSC: Lung squamous cell carcinoma
GSE: Gene expression omnibus series
PPFE: Pleuroparenchymal Fibroelastosis
BSA: Bovine Serum Albumin
IHC: Immunohistochemistry
PMSF: Phenylmethylsulfonyl Fluoride
SDS-PAGE: sodium dodecyl sulfate-polyacrylamide gel electrophoresis
PVDF: Polyvinylidene fluoride
SRB: sulforhodamine B
CCK8: cell counting kit-8
SI: Synergy index
Annexin V-PI: Annexin V-Propidium Iodide
TUNEL: Transferase Mediated Nick End Labeling
DAPI: 4′,6-diamidino-2-phenylindole
DMEM: Dulbecco's Modified Eagle Medium
ECAR: extracellular acidification rate
iTRAQ: Isobaric Tags for Relative and Absolute Quantitatio
CT: Computed Tomography
RECIST: Response Evaluation Criteria In Solid Tumors
DCR: disease control rate
SD: standard deviation
ANOVA: Analysis of Variance
GEO: Gene Expression Omnibus
mRNA: messenger RNA
WT: wild type
EGFR: Epidermal Growth Factor Receptor
PARP: Poly(ADP-ribose) polymerase
ECARs: extracellular acidification rates
ALK: Anaplastic Lymphoma Kinase
ROS1: ROS Proto-Oncogene 1
SD: stable disease
95% CI: 95% Confidence Interval
TRAEs: treatment-related adverse events
KRAS: KRAS Proto-Oncogene
TM4SF1: Transmembrane 4 L Six Family Member1
Akt: Protein Kinase B
mTOR: Mechanistic Target of Rapamycin
EMT: Epithelial-Mesenchymal Transition
PKM2: pyruvate kinase 2
PDK1: pyruvate dehydrogenase kinase 1
