# Supplementary material for: Anlotinib enhances the sensitivity to docetaxel in lung cancer through inhibiting DDR1-mediated glycolytic pathway
Source: Int J Biol Sci. 2026 Aug 24;22(14):7610–27. doi: 10.7150/ijbs.129604 (PMC13617657; doi:10.7150/ijbs.129604)
Supplement: Supplementary file 1 [file ijbsv22p7610s1.pdf]

Anlotinib enhances the sensitivity to docetaxel in lung cancer through inhibiting DDR1-mediated glycolytic pathway

Xueqin Chen[1,2,3#], Juan Shen[2#], Zhifei Xu[4#], Bo Xu[4#], Jie Huang[2], Jingjing Jiang[5], Yidan Chen[2], Xin Li[1], Shaoyu Yang[1], Kan Wu[2], Jiaoli Wang[1], Peihua Luo[4,5], Qiaojun He[4,5,6], Shenglin Ma[1,2, 7*], Bing Xia[1,2*] and Bo Yang[8,6*]

[1]Department of Thoracic Oncology, Affiliated Hangzhou First People's Hospital, School of Medicine, Westlake University, Hangzhou, Zhejiang 310006, PR China

[2]Department of Thoracic Oncology, Hangzhou Cancer Hospital, Hangzhou, Zhejiang 310002, PR China

[3]Fourth Clinical Medical College, Zhejiang Chinese Medical University, Hangzhou, Zhejiang 310053, PR China

[4]Center for Drug Safety Evaluation and Research of Zhejiang University, College of Pharmaceutical Sciences, Zhejiang University, Hangzhou, Zhejiang 310058, PR China

[5]Innovation Institute for Artificial Intelligence in Medicine of Zhejiang University, Hangzhou, Zhejiang 310018, PR China

[6]School of Medicine, Hangzhou City University, Hangzhou, Zhejiang 310015, PR China

[7]School of Medicine, Westlake University, Hangzhou, Zhejiang 310024, PR China

[8]Institute of Pharmacology & Toxicology, College of Pharmaceutical Sciences, Zhejiang University, Hangzhou, Zhejiang 310058, PR China

Running Title: **Anlotinib enhances sensitivity to docetaxel in lung cancer**

[#]Xueqin Chen, Juan Shen, Zhifei Xu and Bo Xu contributed equally to this work

[*]**Corresponding authors.**

E-mail addresses: mashenglin@hospital.westlake.edu.cn (Shenglin Ma), xiabing@hospital.westlake.edu.cn (Bing Xia), yang924@zju.edu.cn (Bo Yang)

Supplementary material includes 4 supplementary figures and legends as well as 2 supplementary tables.

Supplementary Figure 1

A

Patient Number	Gender	Age (y)	Histology	Stage	Gene status	PD-L1 status	Chemotherapy	PFS of docetaxel (Days)
1	Male	61	SCC	IV	N/A	N/A	Pemetrexed/cisplatin	428
2	Male	73	SCC	IIIB	N/A	N/A	Pemetrexed/cisplatin	42
3	Male	64	SCC	IV	Negative	Positive	Paclitaxel/carboplatin	62
4	Male	68	SCC	IV	N/A	Positive	Paclitaxel/cisplatin	66
5	Male	58	SCC	IIIB	N/A	N/A	Paclitaxel/carboplatin	242
6	Male	69	SCC	IV	Negative	N/A	Pemetrexed/carboplatin/sugemalimab	134
7	Male	73	SCC	IIIB	N/A	Negative	Paclitaxel/cisplatin/sugemalimab	58
8	Male	72	SCC	IV	Negative	Positive	Paclitaxel/carboplatin/tislelizumab	340
9	Male	62	LUAD	IV	KRAS mutation	N/A	Pemetrexed/carboplatin/sugemalimab	151
10	Female	55	LUAD	IV	EGFR mutation	N/A	Paclitaxel/carboplatin	27
11	Male	70	LUAD	IV	Negative	Negative	Pemetrexed/carboplatin/sintilimab	125
12	Male	68	SCC	IV	N/A	N/A	Paclitaxel/carboplatin	68

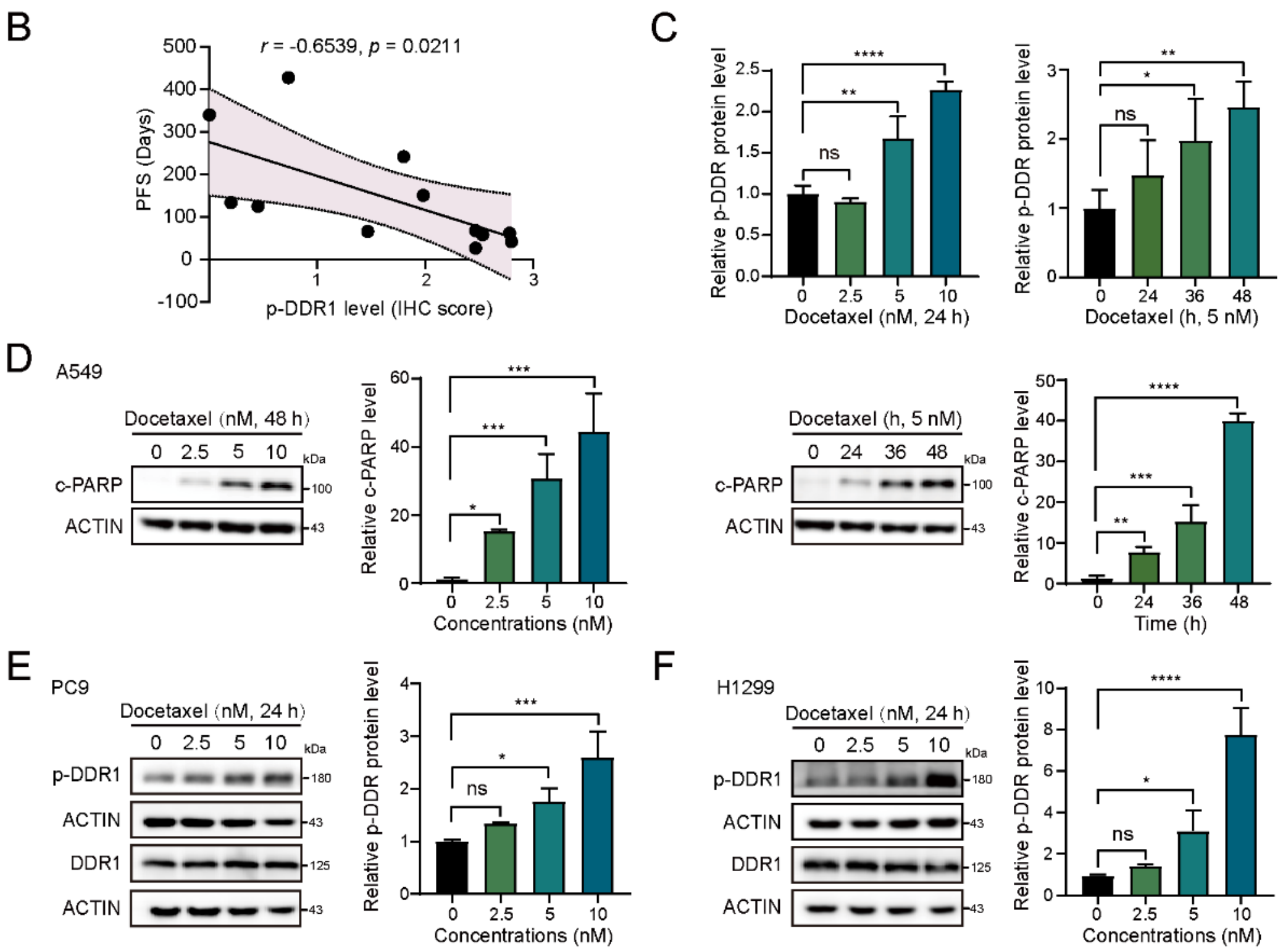

Figure S1. Apoptosis and DDR1 phosphorylation are induced by docetaxel in lung cancer cells, related to Figure 1

A. The baseline characteristics of 12 patients in Figure 1C. SCC indicates Squamous Cell Carcinoma. LUAD indicates Lung Adenocarcinoma. **B.** Correlation between PFS and the levels of p-DDR1 in NSCLC patients prior to docetaxel was calculated using Pearson correlation test. **C.** Quantification of Figure 1E. The time- and concentration-dependent effect used ordinary one-way ANOVA with Dunnett's multiple comparisons

test. ns indicates no significance; *, $p < 0.05$; **, $p < 0.01$ and ****, $p < 0.0001$. **D.** Western blotting showed that the expression of c-PARP increased in a concentration- and time-dependent manner in A549 and quantification of 3 independent experiments. The time- and concentration-dependent effect used ordinary one-way ANOVA with Fisher's LSD test. *, $p < 0.05$; **, $p < 0.01$; ***, $p < 0.001$ and ****, $p < 0.0001$. **E and F.** Western blotting assay showed the expression of p-DDR1 in PC9 cells (E) and H1299 cells (F) treated in a concentration-dependent manner and quantification of 3 independent experiments. The data were analyzed by ordinary one-way ANOVA with Dunnett's multiple comparisons test. ns indicates no significance; *, $p < 0.05$; ***, $p < 0.001$ and ****, $p < 0.0001$.

Supplementary Figure 2

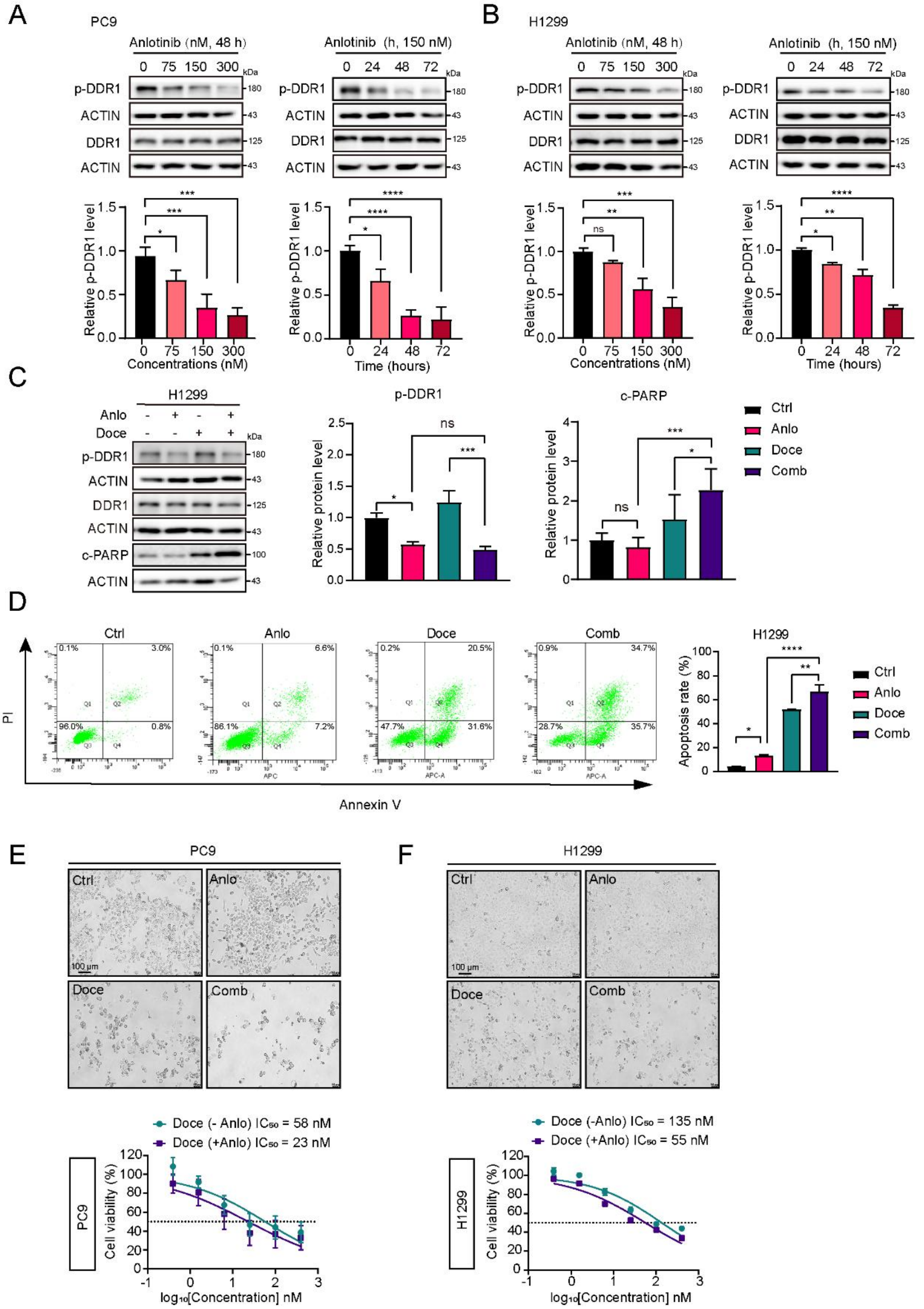

Figure S2. Anlotinib increases the sensitivity to docetaxel by inhibiting DDR1 in H1299 and PC9, related to Figure. 2

A and B. Western blotting assay showed the expression of p-DDR1 in PC9 cells (A) and H1299 cells (B) treated in a concentration- and time- dependent manner and quantification of 3 independent experiments. The time- and concentration-effects were analyzed by ordinary one-way ANOVA with Dunnett's multiple comparisons test. ns indicates no significance; *, $p < 0.05$; **, $p < 0.01$; ***, $p < 0.001$ and ****, $p < 0.0001$. **C.** The levels of p-DDR1 andc-PARP in H1299 cells treated with 150 nM anlotinib and/or 5 nM docetaxel for 48 h and quantification of 3 independent experiments. The data were analyzed by ordinary one-way ANOVA with Tukey's multiple comparisons test. ns indicates no significance; *, $p < 0.05$ and ***, $p < 0.001$. **D.** Flow cytometry analyzed apoptosis with Annexin V-PI staining in H1299 cells treated with 150 nM anlotinib and/or 5 nM docetaxel for 48 h and quantification of 3 independent experiments. The data were analyzed by ordinary one-way ANOVA with Tukey's multiple comparisons test. *, $p < 0.05$; **, $p < 0.01$ and ****, $p < 0.0001$. **E and F.** Morphology of PC9 cells (E) and H1299 cells (F) treated with 150 nM anlotinib and/or 5 nM docetaxel for 48 h was examined by optical microscopy and cell viability following treatment with gradient-diluted docetaxel and/or 150 nM anlotinib. Original magnification, 100 ×; scale bars, 100 μm.

Supplementary Figure 3

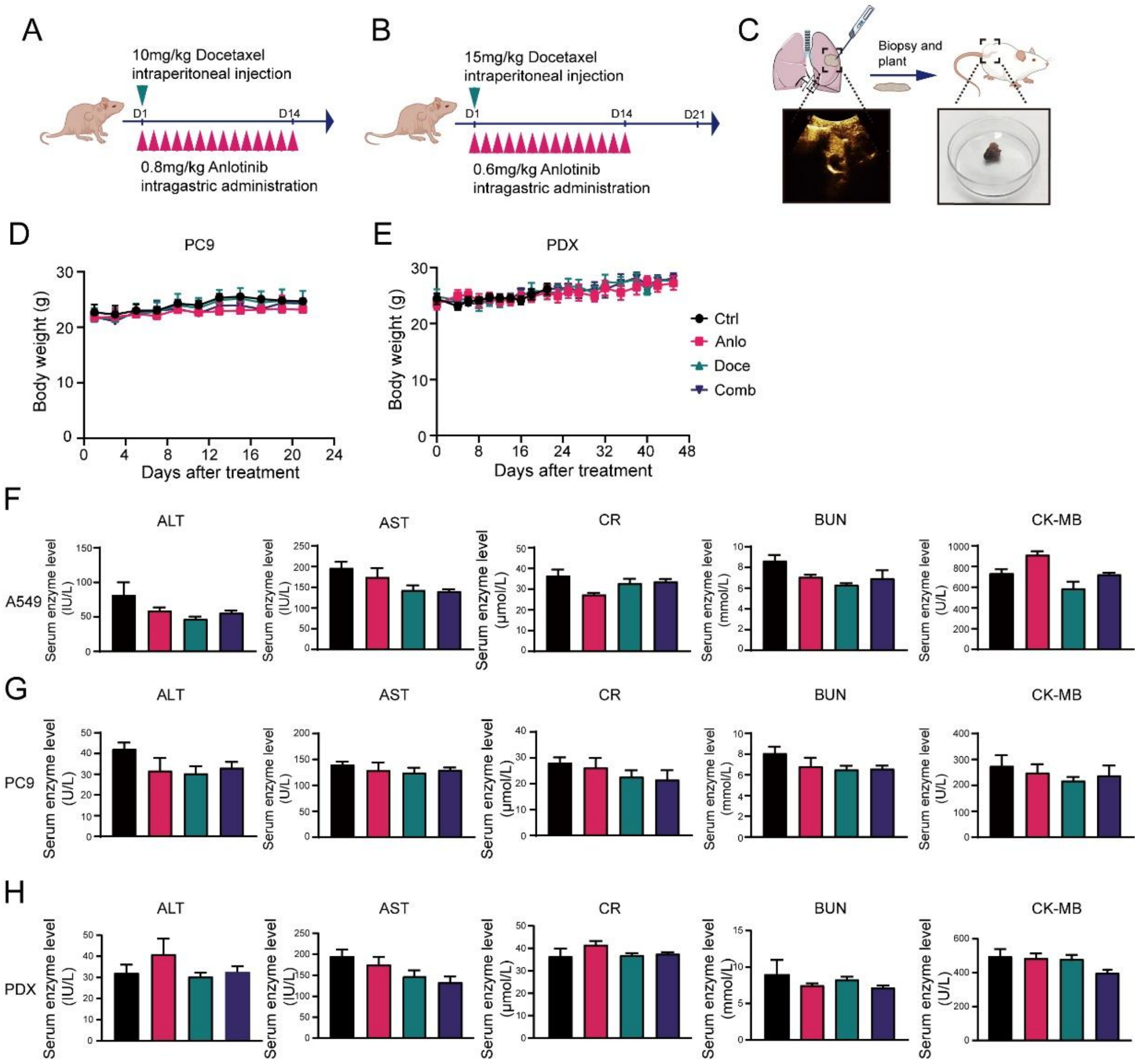

Figure S3. The safety of combination with anlotinib and docetaxel in lung cell- and patient-derived tumor xenografts, related to Figure 3

A and B. Schematic drawing of A549 (A) and PC9 (B) xenografts administered with docetaxel and anlotinib. **C.** Schematic diagram of PDX is shown. **D and E.** The body weight of PC9 (D) and PDX (E) xenograft was measured every two days for each group. **F-H.** The levels of serum enzymes (ALT, AST, CR, BUN, CK-MB) were displayed when the A549 (F), PC9 (G) and PDX (H) xenograft-bearing mice were euthanized.

ALT indicates alanine aminotransferase; AST indicates aspartate aminotransferase; CR indicates creatinine; BUN indicates blood urea nitrogen and CK-MB indicates creatine kinase-MB isoenzyme

Supplementary Figure 4

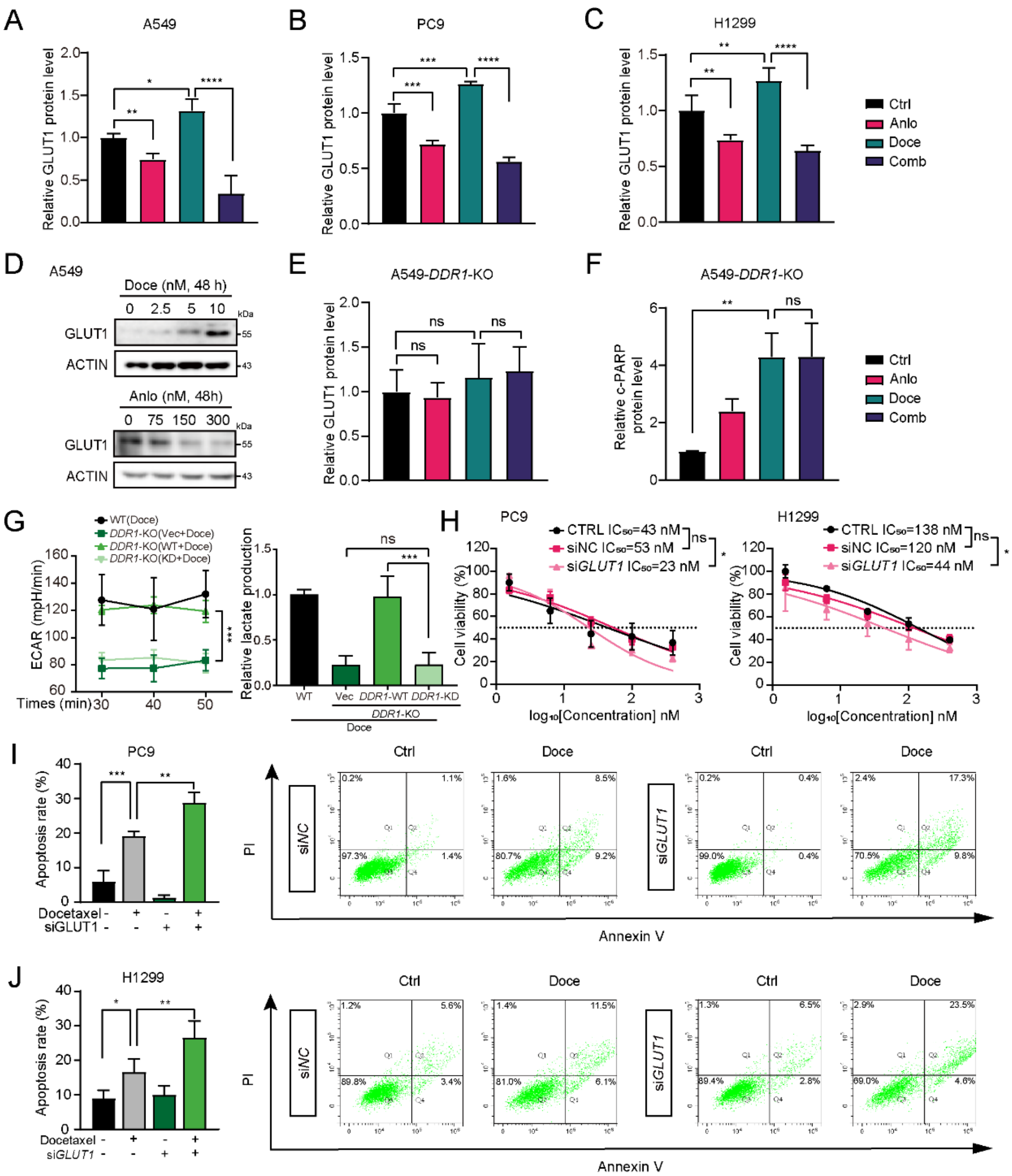

Figure S4. Anlotinib enhances the sensitivity to docetaxel in NSCLC via inhibiting DDR1-mediated glycolysis pathway, related to Figure 5

A-C. Quantification of GLUT1 in A549 cells (A), PC9 cells (B) and H1299 cells (C) treated with 150 nM anlotinib and/or 5 nM docetaxel for 48 h. The data were analyzed by ordinary one-way ANOVA with Tukey's multiple comparisons test, data are presented as mean ± SD of three independent experiments. *, $p < 0.05$; **, $p < 0.01$;

***, $p < 0.001$; and ****, $p < 0.0001$. **D.** The expression of GLUT1 in A549 cells treated with various concentrations of anlotinib or docetaxel. **E and F.** Quantification of Figure 5F; data are present as mean ± SD of three independent experiments. The data were analyzed by ordinary one-way ANOVA with Tukey's multiple comparisons test. ns indicates no significance; **, $p < 0.01$. **G.** ECAR and measurement of lactate production of A549 and A549 *DDR1*-KO cells transfected with empty vector (Vec), wild-type *DDR1* (*DDR1* WT), or kinase-dead *DDR1* K618A mutant (*DDR1* KD) treated with or without 5 nM docetaxel for 48 h. The data were analyzed by ordinary two-way ANOVA with Tukey's multiple comparisons test and Student's t-test. ns indicates no significance and ***, $p < 0.001$. **H.** Dose-response curves of docetaxel for 48 h upon PC9 and H1299 cells with or without *GLUT1* knockdown. The data were analyzed by two-way ANOVA with Tukey's multiple comparisons test (n = 3). Error bars represent means ± SD. ns indicates no significance and *, $p < 0.05$. **I and J.** Apoptosis was analyzed by flow cytometry with Annexin V-PI staining in PC9 cells (I) and H1299 cells (J) with or without *GLUT1* knockdown treated with 5 nM docetaxel for 48 h, and quantification of 3 independent experiments. The data were analyzed by ordinary one-way ANOVA with Tukey's multiple comparisons test; *, $p < 0.05$; **, $p < 0.01$ and ***, $p < 0.001$.

ECAR indicates extracellular acidification rate

Table S1. The demography and baseline characteristics

Characteristics	All treated patients (n=45)
Age, median(range), years	61 (37-74)
Male, No. (%)	35 (77.8)
ECOG performance status, No. (%)	
0	8 (17.8)
1	37 (82.2)
Histology, No. (%)	
Adenocarcinoma	30 (66.7)
Squamous cell carcinoma	12 (26.7)
Other	3 (6.7)
Stage, No. (%)	
IIIb	2 (4.4)
IVa	27 (60.0)
IVb	16 (35.6)
Gene status, No. (%)	
EGFR/ALK/ROS1 negative	28 (62.2)
EGFR mutation only	9 (20.0)
ALK fusion only	3 (6.7)
Unknown	5 (11.1)
PD-L1 status[a], No. (%)	
Negative	11 (24.4)
Positive	8 (17.8)
Unknown	26 (57.8)
Prior systemic treatment regimens, No. (%)	
Targeted therapy	14 (31.1)
Immunotherapy	17 (37.8)
Antiangiogenic therapy	14 (31.1)

[a]PD-L1 positivity was defined as tumor proportion score (TPS) ≥ 1% using PD-L1 immunohistochemistry 22C3 assay (Agilent Technologies).

ECOG: eastern cooperative oncology group

Table S2. Summary of treatment-related adverse events (TRAEs)

Adverse events, No. (%)	Grade (1-2)	Grade (3-4)	All grades
HFSR[a]	9 (20.0)	4 (8.9)	13 (28.9)
Hypertension	7 (15.6)	5 (11.1)	12 (26.7)
Hemorrhage[b]	9 (20.0)		9 (20.0)
Stomatitis	7 (15.6)	2 (4.4)	9 (20.0)
Leucopenia	5 (11.1)	3 (6.7)	8 (17.8)
Anaemia	7 (15.6)		7 (15.6)
Thrombocytopenia	5 (11.1)		5 (11.1)
Electrolyte disturbance[c]	3 (6.7)	3 (6.7)	5 (11.1)
Fatigue	1 (2.2)	2 (4.4)	3 (6.7)
Bronchopneumonia	1 (2.2)	2(4.4)	3 (6.7)
Hepatic enzyme increased	3 (6.7)		3 (6.7)
Exanthema	3 (6.7)		3 (6.7)
Hypertriglyceridemia	3 (6.7)		3 (6.7)
Hyperglycemia	3 (6.7)		3 (6.7)
Hypercholesteremia	2 (4.4)		2 (4.4)
Blood creatinine increased	2 (4.4)		2 (4.4)
Hypoproteinemia	2 (4.4)		2 (4.4)
Blood bilirubin increased	1 (2.2)		1 (2.2)
Itching	1 (2.2)		1 (2.2)
Decreased appetite	1 (2.2)		1 (2.2)
Acute coronary syndrome		1 (2.2)	1 (2.2)
Proteinuria	1 (2.2)		1 (2.2)
Nephrotic syndrome	1 (2.2)		1 (2.2)
Hyperuricemia	1 (2.2)		1 (2.2)
Cerebrovascular ischemia	1 (2.2)		1 (2.2)

[a]HFSR: hand-foot skin reaction

[b]Hemorrhage: hemoptysis, hematuria, hematochezia

[c]Electrolyte disturbance: hyponatraemia, hypokalaemia
